# Assessing the Fate of Benzophenone-Type UV Filters and Transformation Products during Soil Aquifer Treatment: The Biofilm Compartment as Bioaccumulator and Biodegrader in Porous Media

**DOI:** 10.1021/acs.est.3c08465

**Published:** 2024-03-11

**Authors:** Sònia Jou-Claus, Paula Rodríguez-Escales, Lurdes Martínez-Landa, M. Silvia Diaz-Cruz, Jesús Carrera, Adrià Sunyer-Caldú, Gerard Quintana, Cristina Valhondo

**Affiliations:** † Dept. of Civil and Environmental Engineering, Universitat Politècnica de Catalunya, Jordi Girona 1-3, 08034 Barcelona, Spain; ‡ Associated Unit: Hydrogeology Group (UPC−CSIC), Universitat Politècnica de Catalunya, Jordi Girona 1-3, 08034 Barcelona, Spain; § Institute of Environmental Assessment and Water Research Severo Ochoa Excellence Center, Spanish National Research Council (IDAEA-CSIC), Barcelona 08034, Spain; ∥ Department of Environmental Science (ACES, Exposure & Effects), Science for Life Laboratory, Stockholm University, Stockholm 106 91, Sweden

**Keywords:** benzophenone-3, oxybenzone, managed aquifer recharge, reactive barriers, redox potential, sorption, biodegradation, biofilm

## Abstract

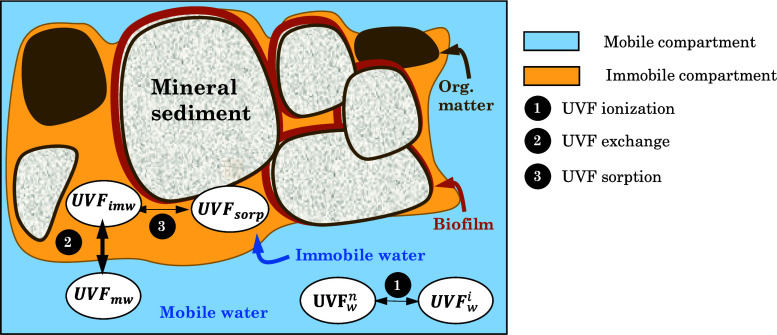

The fate of selected UV filters (UVFs) was investigated in two soil aquifer treatment (SAT) systems, one supplemented with a reactive barrier containing clay and vegetable compost and the other as a traditional SAT reference system. We monitored benzophenone-3 (BP-3) and its transformation products (TPs), including benzophenone-1 (BP-1), 4,4′-dihydroxybenzophenone (4DHB), 4-hydroxybenzophenone (4HB), and 2,2′-dihydroxy-4-methoxybenzophenone (DHMB), along with benzophenone-4 (BP-4) and avobenzone (AVO) in all involved compartments (water, aquifer sediments, and biofilm). The reactive barrier, which enhances biochemical activity and biofilm development, improved the removal of all detected UVFs in water samples. Among monitored UVFs, only 4HB, BP-4, and AVO were detected in sediment and biofilm samples. But the overall retained amounts were several orders of magnitude larger than those dissolved. These amounts were quantitatively reproduced with a specifically developed simple analytical model that consists of a mobile compartment and an immobile compartment. Retention and degradation are restricted to the immobile water compartment, where biofilm absorption was simulated with well-known compound-specific *K*_ow_ values. The fact that the model reproduced observations, including metabolites detected in the biofilm but not in the (mobile) water samples, supports its validity. The results imply that accumulation ensures significant biodegradation even if the degradation rates are very low and suggest that our experimental findings for UVFs and TPs can be extended to other hydrophobic compounds. Biofilms act as accumulators and biodegraders of hydrophobic compounds.

## Abstract 2

UV filters tend to accumulate preferentially in the biofilm and immobile aquifer sediment compartments of SAT systems, and their degradation is enhanced in the presence of reactive barriers.

## 1 Introduction

Ultraviolet filters (UVFs) are widely used in numerous personal care and hygiene products.^1^ Benzophenones, such as benzophenone-3 (BP-3), benzophenone-4 (BP-4), and avobenzone (AVO) are among the most used organic UVFs. These compounds, as other UVFs, are characterized as photostable and lipophilic,^2^ thus bioaccumulating and biomagnifying along the trophic web.^1,3^ Benzophenones and their derivatives have been reported as endocrine disruptors.^4^ They cause adverse effects in fish and rodents’ fecundity,^5^ neurotoxicity, cytotoxicity, and Hirschsprung disease.^6^ In humans, they have been associated with estrogen-dependent diseases such as endometriosis.^7^ Despite their lipophilic nature, they have been detected in all types of water: surface, seawater,^8^ wastewater,^9^ tap water,^10^ and groundwater.^11^ UVFs have also been found in solid environmental matrices such sewage sludge,^12^ sediments,^13^ and biota (fish, marine mammals, birds, and invertebrates).^14−16^

Managed aquifer recharge (MAR) is considered a potential source of UVFs into groundwater bodies.^17^ This is especially true when recharged water is the treated effluent of a wastewater treatment plant (WWTP). While MAR contributes to augmenting water resources, regulatory concerns have arisen regarding the possibility of aquifer contamination.^18^ Nonetheless, MAR can be integrated as a tertiary treatment process in conjunction with a WWTP, termed Soil Aquifer Treatment (SAT), since infiltration through the porous media promotes the natural attenuation of many recalcitrant compounds. Valhondo et al. proposed the installation of reactive barriers at the bottom of infiltration ponds to further enhance degradation and sorption processes during SAT.^19^ This reactive barrier consists of a mixture of natural materials providing a range of sorption sites (neutral, cationic, and anionic) and enhancing biodegradation by the release of labile organic carbon, which in turn promotes a broad spectrum of reduction–oxidation (redox) states, thereby expanding the pathways for degradation and the removal of contaminants of emerging concern (CECs).^20^

Sorption of UVFs, as other CECs, is governed by their affinity to the immobile organic phases (as characterized by octanol–water partition constant, *K*_ow_, or organic carbon–water partition constant, *K*_oc_) and, when ionized, by interactions with charged solid surfaces.^21−23^ In turn, these sorption mechanisms are affected by numerous chemical parameters of the solution (ionic strength, pH, concentrations of competing ions) and the solid surfaces (their composition, surface charge, and the organic matter age).^24^ Ionizable organic molecules, with p*K*_a_ within the experimental pH range, may change their ionic state and therefore their sorption mechanism. In groundwater, the pH usually ranges between 6 and 8. This is relevant for some UVFs, such as benzophenones, whose p*K*_a_s are mostly within this range (Table 1), causing both neutral and ionic forms to coexist.

**Table 1:** Physicochemical Properties of UVFs (from ChemSpider Database)^32^

abbreviation	other names	CAS	MW (g mol^–1^)	log *K*_ow_	log *K*_oc_	p*K*_a_	solubility (g L^–1^, H_2_O)
BP-3	oxybenzone; 2-hydroxy-4-methoxybenzophenone	131-57-7	228.24	3.79	3.10	7.56	0.21
BP-4	5-benzoyl-4-hydroxy-2-methoxybenzene sulfonic acid; HMBS; sulisobenzone	4065-45-6	308.31	0.37	1.96	–2.42	0.65
AVO	1-(4-*tert*-butylphenyl)-3-(4-methoxyphenyl)propane-1,3-dione	70356-09-1	310.39	4.51	3.23	6.55	0.0015
BP-1	2,4-dihydroxybenzophenone	131-56-6	214.22	2.96	3.46	7.09	0.39
DHMB/ (BP-8)	2,2′-dihydroxy-4-methoxybenzophenone; benzophenone-8; dioxybenzone	131-53-3	224.24	3.82	3.32	6.78	0.052
4HB	4-hydroxybenzophenone	1137-42-4	198.22	3.02	3.24	7.85	0.41
4DHB	4,4′-dihydroxybenzophenone	611-99-4	214.22	2.19	3.45	7.55	0.60

UVFs biodegrade in groundwater under aerobic and anaerobic conditions, but mainly by cometabolism.^25−27^ Therefore, the presence of labile organic carbon enhances their biodegradation. The biodegradation pathway, determined by the redox state and the electron acceptor availability, is a key parameter controlling the type of transformation product (TP).^25^ Biodegradation occurs mainly inside the biofilms, which are an assemblage of microorganisms comprising microbial species attached to a surface and encased in a self-synthesized matrix with water and extracellular polymeric substances (EPS). They can act as active sorbents to organic compounds in porous media.^28,29^ EPS is both positively and negatively charged, favoring the retention of anionic and cationic species. There are also lipids that promote the retention of lipophilic compounds. Although there is some evidence of organic micropollutants retention in biofilm in WWTPs,^22^ there is no experimental evidence about their retention in porous media, and neither regarding biotransformation and retention mechanisms with respect to changes in redox conditions promoted by the reactive barriers.

This study aims to assess, through both experimental methods and a numerical model, the role of biofilm in the sorption and degradation processes of specific benzophenone-type UV filters found in a real WWTP effluent. Two soil aquifer treatment systems were utilized for this investigation, with one of them incorporating a reactive barrier at the infiltration pond. The selected UVFs were BP-3 and its main TPs, benzophenone-1 (BP-1), 4,4′-dihydroxybenzophenone (4DHB), 4-hydroxybenzophenone (4HB), and 2,2′-dihydroxy-4-methoxybenzophenone (DHMB), BP-4, and AVO. Analyzed UVFs samples comprised all of the phases present in the SAT system: water, aquifer sediments, and biofilm.

## 2 Materials and Methods

### 2.1 Soil Aquifer Treatment Site and Experimental Design

**Figure 1:**
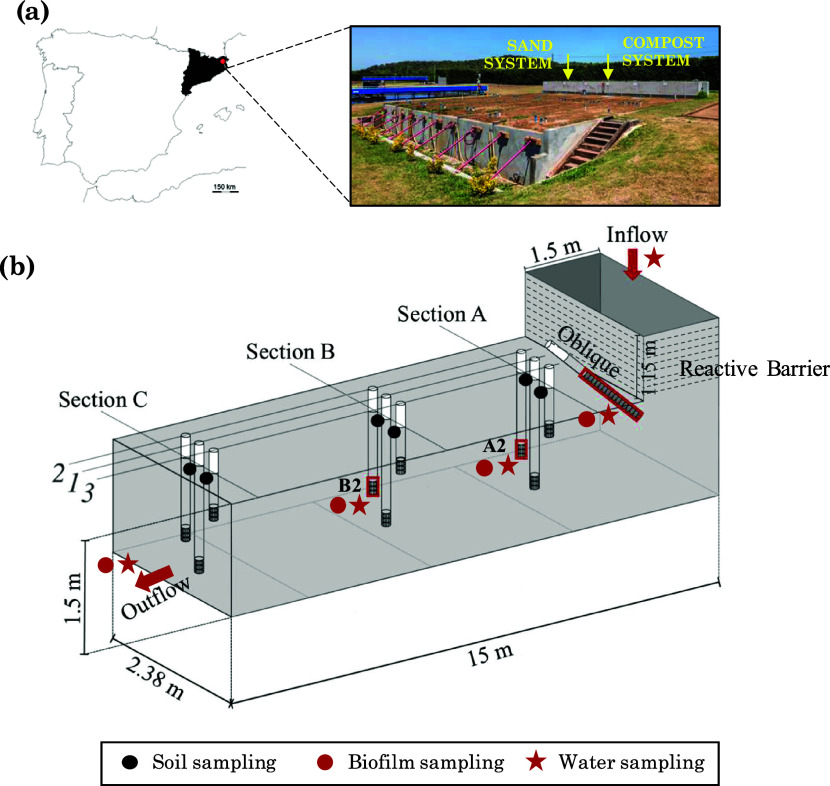
Soil aquifer treatment (SAT) pilot system at Palamós (Girona, Spain). (a) Location and picture of the facility with the six systems. (b) Scheme description of each system. Black dots represent the sand sampling points, whereas the red ones represent the biofilm and water sampling points.

Two pilot SAT systems were tested at the Palamós WWTP (Girona, Spain). Each consists of a constructed aquifer coupled to an infiltration basin fed with a WWTP secondary effluent (Figure 1). One of the two systems, referred to as “CT” (compost-treatment), contained a 1 m thick reactive barrier that had been installed 2 years before the UVF experiment. The barrier was made up of sand (0.15 to 0.4 mm particle sand to provide structure, 49% in volume), vegetal compost (from tree pruning to provide sorption sites for lipophilic compounds and release DOC, 49%), and clay (providing cation sorption sites 2%). The other system is denoted “ST” (sand-treatment) as it reflects the conventional SAT systems, consisting solely of sand. The resulting overall porosities were 0.38 for ST and 0.48 for CT.

Both systems were equipped with PVC piezometers for monitoring along the flow path (Figure 1): 9 in the aquifer (sections A-C in Figure 1), and a fully screened inclined piezometer at the base of the barrier (oblique piezometer, referred to as “O” hereafter) to collect water exiting the barrier. The outflow was integrated and collected by a discharge pipe installed at the base of the aquifer. Piezometers were sampled with submersible centrifugal pumps (nontoxic ABS plastics, Stainless-steel impeller, and silicone delivery pipe). Groundwater level, electrical conductivity (EC), and temperature were regularly monitored. A detailed description of the pilot systems can be found in Valhondo et al., 2020.^30^

Our experiment consisted of the continuous monitoring of UVFs and TPs (Table 1) along the SAT systems (inflow, section A, section B, section C, and outflow (Figure 1)), within the three environmental compartments: water, aquifer sediments, and biofilm. The monitored UVFs were those naturally present in the recharged water, i.e., the secondary treatment effluent (STE) from the Palamós WWTP. UVFs monitoring started after the injection of lithium acetate (LiAc), acting as a tracer of the experiment. The monitoring extended over 86 days, during which UVFs, LiAc, and hydrochemical parameters were meticulously characterized.

Prior to the experiment, the SAT systems were operated for one month, with a flow rate of 1 m^3^/d. The purpose of this pre-experimental phase was to establish a steady state flow and to characterize conservative transport and arrival times at monitoring points (details in S2 in the SI). The same flow conditions were kept during the whole experiment (86 days). The water level at the outlet was set at 135 cm for both systems, resulting in an approximate total residence time in the tanks of around 12 days.

Tracer injection (day 0) consisted of 57 L of water from the STE spiked with LiAc at 17.8 mg/L in the infiltration basins of the ST and CT systems. LiAc was selected because Li^+^ acted as a sorbing tracer and Ac^–^ as a source of easily degradable organic carbon, favoring redox processes, as well as UVF degradation. Li^+^ was an adequate tracer because: (1) it did not interact with UVFs determinations as colorimetric tracers or bromide;^31^ (2) it was absent in the STE, and, (3) it was nondegradable. Water, sediments, and biofilm samples were collected at scheduled times during the experiment (S2 in the SI).

### 2.2 Sampling at the Different Matrixes and Analytical Methods

#### 2.2.1 Groundwater Sampling and Hydrochemistry Characterization

Water samples were collected from inflow (STE), piezometers (O, A2, B2, and C2), and outflow of the two SAT systems, after purging the piezometers using drive pumps. Physicochemical parameters (EC, pH, redox potential (*Eh*), and temperature) were measured *in situ* with a multiparameter probe (YSI, Inc. Yellow Springs, OH). Alkalinity was measured also *in situ* with a test kit (Merck Millipore, Darmstadt, Germany). Water hydrochemistry characterization consisted in the analysis of dissolved organic carbon (DOC), NH_4_^+^, major cations, and anions (sampling and analytical details in S3). Samples for UVFs analysis were collected in amber glass bottles of 150 mL, immediately frozen, and kept in the dark to prevent photo- and biodegradation. The analyzed UVFs are listed in Table 1, and analytical determination is described in Section 2.3.

#### 2.2.2 Aquifer Sediments and Biofilm Characterization

Nine sediment samples were collected from each SAT system at different locations (see Figure 1) and at different experimental times (see S2 in the SI). Samples were taken at 55 cm depth, which was the same depth as the screened interval of the piezometer. Sampling was done using a 110 cm long and 1 cm wide drill. The sediment samples were collected to determine the UVFs concentrations (Section 2.3) as well as the fraction of sedimentary organic carbon (*f*_oc_) (S6 of SI).

Biofilm in the SAT systems was characterized using biotraps installed in the piezometers (O, A2, and B2) at 55 cm depth of each SAT system (Figure 1). Biotraps consisted of sandbags packed into a protective plastic mesh. The siliceous sand was the same as the aquifer and was previously muffled for 5 h at 600 °C to remove all sedimentary organic matter (SOM) and to ensure that any observed retention of UVFs in the biotraps was solely attributed to the biofilm, as little interaction with the silica sand was anticipated.^28^ Biotraps were installed one month before the test to promote bacterial growth and biofilm formation. UVFs concentration, bacterial density, and EPS (as an amount of biofilm measurement) were determined in the biotrap samples (see Section 2.3 and S6 in the SI).

#### 2.2.4 UVFs Analysis: Water, Sediments, and Biofilm

UVFs determination in the sediments and water matrices followed previously developed methods.^33,34^ However, a new methodology was developed and validated for biofilm analysis. The method consisted of extraction and purification using QuEChERS and further analysis by liquid chromatography coupled with tandem mass spectrometry (LC-MS/MS). Separation and quantification of the target analytes were performed by high-performance liquid chromatography in a Hibar Purosher STAR HR R-18 (50 mm × 2.0 mm, 5 μm) column using a Symbiosis Pico instrument from Spark Holland (Emmen, The Netherlands) attached to a 4000 QTRAP mass spectrometer from Applied Biosystems-Sciex (Foster City). The achieved limits of detection (LODs) ranged between 0.18 and 0.87 ng/g of dw, and the limits of quantification (LOQs) ranged between 0.60 and 2.89 ng/g of dw, showing the method’s good sensitivity. The relative standard deviation (RSD) values were below or equal to 20%, indicating good precision. Accuracy was evaluated by the recovery rates of each standard spiked in a representative mixture of biofilms at two concentration levels. Recovery rates at 5 ng/g dw spike level were between 72.48 and 131.2%, and at 100 ng/g dw, recovery rates were between 86.4 and 122.8%. Further details about these analytical methods are provided in the SI (S6.4 section), including the instrumental analysis, method validation, and QA/QC for the biofilm analysis.

### 2.3 Conceptual and Numerical Model of the UVFs Fate at the SAT Systems

**Figure 2:**
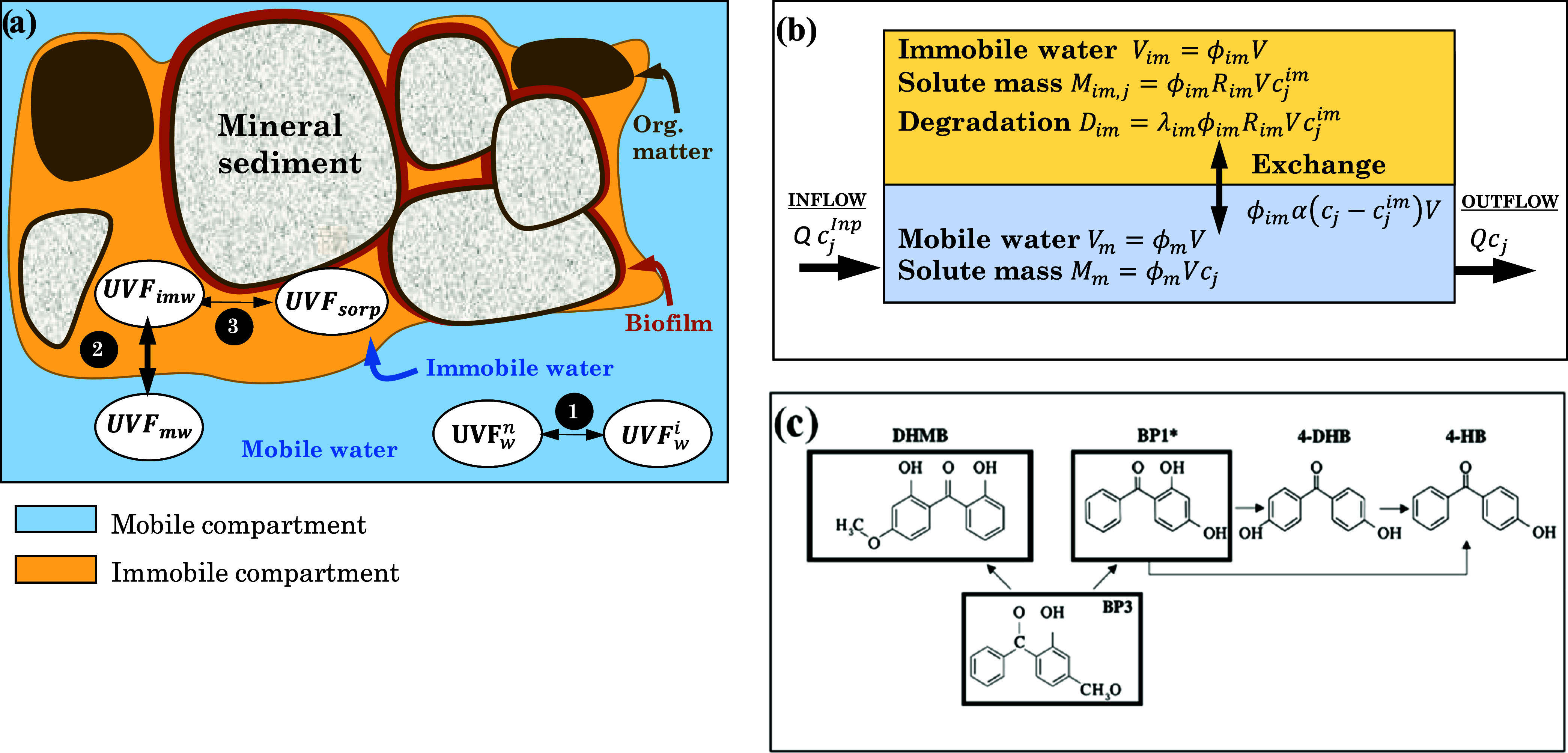
(a) Conceptual representation of UVFs partitioning processes, where (1) refers to the ionization, exchange between neutral (n) and ionic (i) forms, controlled by pH and p*K*_a_; (2) exchange between mobile (m) and immobile waters (im), controlled by molecular diffusion; and (3) retention into the sediment characterized by ionic interactions or by affinity to organic matter. (b) Two-compartment model adopted for equilibrium and mass balance calculations and (c) biodegradation pathways of BP-3 and transformation products. * Compounds also formed in anoxic conditions.

The fate of UVFs and most pollutants in porous media is mostly controlled by advection/mixing-dispersion and by sorption and degradation processes. Inside the biofilm, the most important processes are the different retention mechanisms, diffusion, as well as degradation^35^ (Figure 2a).

For a semiquantitative interpretation of observations and to evaluate the role of the processes involved in the biofilm retention, we built a simple (in that processes are represented by simplified equations) model yet complex (in that numerous variables are involved). The model simplifies the SAT system by representing it as a single cell (or box) with two compartments: the mobile one and the immobile (Figure 2b), in a similar and simplified way as multi-rate mass transfer model.^36^ The immobile compartment represents water in biofilms, microorganisms, and extracellular biological material, sedimentary organic matter, and isolated pores. Therefore, sorption (both absorption into organic and biological solids and adsorption onto mineral surfaces and charged organic matter) occurs primarily in the immobile compartment. Similar to previous works,^37^ it is also assumed that microbial communities responsible for degradation reactions live in biofilms and they are mature so that degradation is limited by the concentration of the compound and can be taken as first order. This assumption was reasonable in our system since the SAT systems were run for more than 2 years. Instead, the mobile compartment represents free-flowing water so that sorption and degradation are neglected.

These compartments are characterized by (1) their exchange rate, α, inverse of the mean residence time in the immobile water compartment (promoted by diffusion through the immobile compartment), (2) the mobile and immobile porosities, ϕ_m_ and ϕ_im_, respectively, both referred to the total volume of the medium, so that the total porosity (assumed to be 0.25) is ϕ = ϕ_m_ + ϕ_im_, (3) sorption properties (*K*_ow_ for absorption into lipophilic substances present in biofilms, *K*_oc_ for absorption into aquifer organic matter, and *K*_d_ as a lumped parameter for adsorption onto ionic surfaces), blended into the retardation factor, *R*_im,*j*_ (ratio of total, sorbed plus dissolved, to dissolved mass in the immobile compartment, computed from partition parameters as discussed in the SI), and (4) degradation rates λ_*p*,*j*_ [T^–1^] (of parents *p* to daughters *j*). The latter describe the degradation pathways, which are complex and dependent on redox conditions. The model cannot reproduce redox conditions, which would require multiple immobile zones (the most reducing conditions are reached in the least accessible portions of the medium). Therefore, a simplified degradation network, using only the analyzed species and neglecting redox state, has been adopted^25,38,39^ (Figure 2c). This network is further discussed in Section 3.2.

The resulting model, equilibrium, and mass balance calculations as well as all used parameters are described in S8 in the SI. Sorbed concentrations (*S*_*j*_^im^) were derived from the observed dissolved concentrations as given by
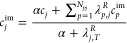
1a
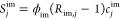
1bwhere *c*_*j*_ and *c*_*j*_^im^ (M V^–1^) are the concentrations of the *j*-th solute in the mobile and immobile compartments, respectively, *S*_*j*_^im^ (M V^–1^) is the mass retained in solid phases per unit volume of sediments, and λ_*p*,*j*_^*R*^ (T^–1^) is the effective degradation rate of each parent defined as
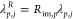
2which depends on λ_*j*,*T*_^*R*^ = ∑_*d*=1_^*N*_*dj*_^λ_*j*,*d*_^*R*^, the effective total rate (sum of all degradation paths). These calculations were performed using the mean observed water concentration at piezometers in section B (representative of mean conditions) for *c*_*j*_ in eq 1a and then to obtain *S*_*j*_^im^. The modeled results were compared to the measured retained mass in the sediment samples since they contained both biofilm and sedimentary organic matter. They were calculated by averaging spatial concentrations since results were comparable before and after the acetate injection, besides this, they were considered more representative of the overall aquifer system than those of biotraps (Table S10). The mass balance of BP-3 and TPs (Figure 2) is given by (see the SI for details)
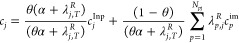
3where θ = *Q*/(*Q* + *V*αϕ_im_) relates the advective flux of solutes (*Q* is the mean flow rate) and the total flux, advective plus mobile immobile (*V* is the volume of sediments), and *c*_*j*_^Inp^ is the input concentration (average of the 15 inflow samples, using half of the detection limit for samples below). This equation was applied sequentially, starting with BP-3, using eq 3 to compute the outflow and (1a) to compute the immobile water concentrations for the computation of daughter compounds in the chain of Figure 2. These outflow concentrations were compared to the average of the 6 outflow samples (see Tables S5–S7 in the SI for the detailed inflow and outflow UVFs concentrations).

## 3 Results and Discussion

### 3.1 Transport and Redox Conditions in the SAT Systems

Groundwater flow and conservative transport in the two SAT systems were comparable, showing similar arrival times of both EC and lithium at the different sampling points (Figure S3a,b and c,d respectively). In the two systems, preferential flow dominated transport through the recharge zone, with a fast-early arrival and a broad range of residence times at the first observation point of the two systems (O-piezometer), similarly to Valhondo et al.^40^ On the other hand, transport in the aquifer was what might be expected from a relatively homogeneous aquifer with moderate dispersion for short transport length (up to A2), but more significant dispersion up to B2 (Figure S3).

**Figure 3:**
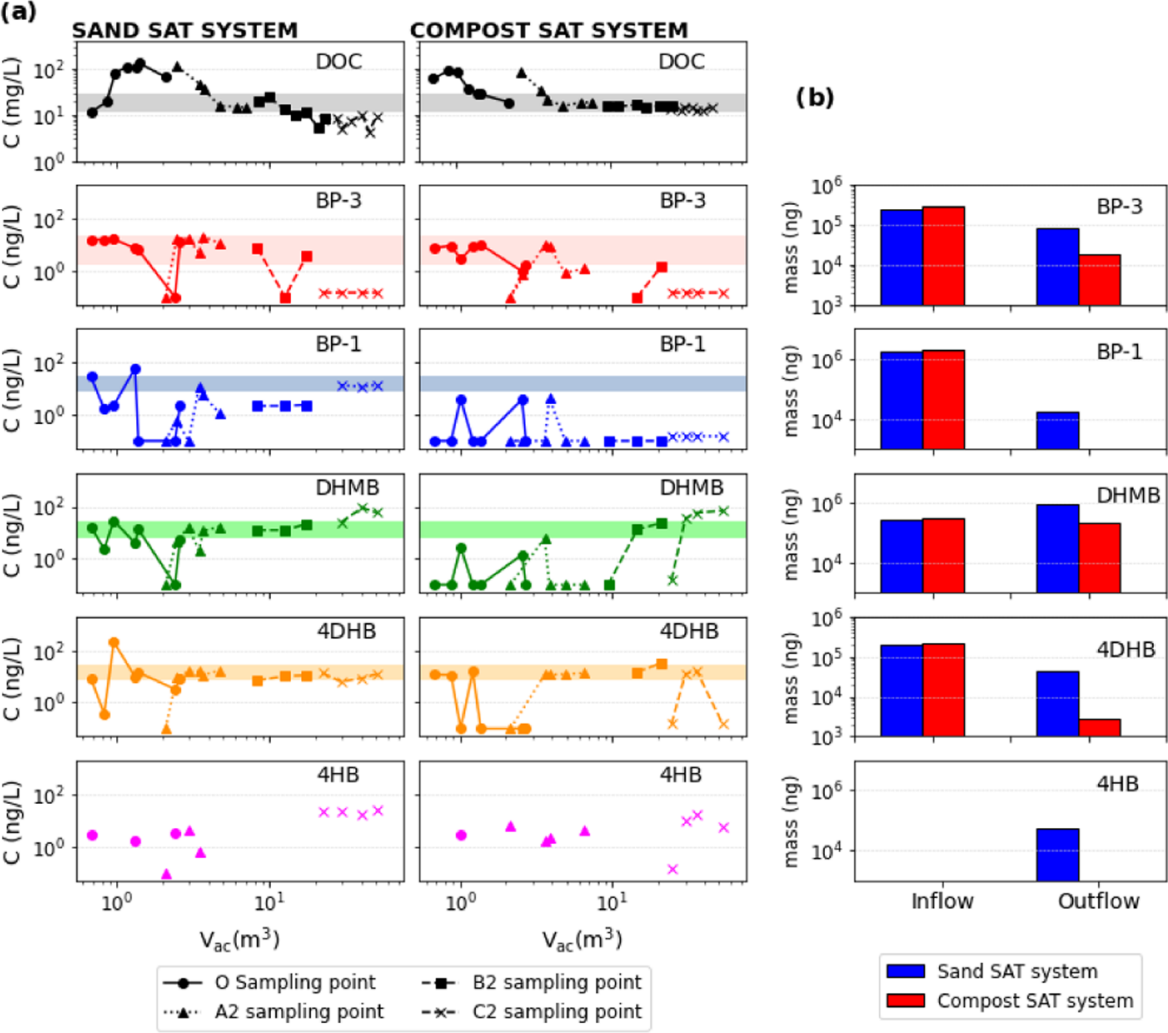
(a) Evolution of the concentration of DOC, BP-3, and TPs at the different sampling points vs the cumulative injected water volume along the two SAT pilot systems. The corresponding volumes for the sampling points are approximately 1(O), 3(A2), and 9(B2) m^3^. The shadow rectangle represents the range of influent concentrations. (b) Mass balance of BP-3 and TPs in the two SAT systems. The “inflow” represents the cumulative mass input during the initial 12 days of the experiment. The “outflow” represents the mass present in the effluent after undergoing the residence time within the SAT system for the same duration.

Redox zonation (*pe* in Figure S3) was largely controlled by the oxidation of DOC and ammonium in the recharged water. The breakthrough curves (BTCs) of DOC (Figures 3 and S3e,f) were similar to those of Li^+^ (Figure S3c,d) in that they displayed a fast peak and a much lower concentration for the CT than for the ST. The main difference between them is that the tails of the DOC BTCs dropped much faster than the tails of Li^+^ BTCs, which we attribute to strong biogeochemical activity in the slow flow and immobile portions, especially in the CT. In fact, DOC was mostly degraded by the time the plume reached the B2 point (fully degraded in the CT). That is, the presence of the reactive barrier enhanced the biological activity and the organic carbon oxidation, which is consistent with observations of Valhondo et al.^30,41,42^ Recall that the reactive barrier had been installed 2 years before the slug injection, and one of its goals was to release DOC to favor reducing conditions. By the end of this 2-year period, it was expected that this release had been largely depleted.^43,44^ Therefore, we attribute the higher degradation in the CT system to a richer bacterial activity as was established in Hellman et al.^45^ The greater reactivity in CT is also supported by the Li^+^ breakthrough curves (Figure S2). The tail of Li^+^ lasts longer than that of CE, and more so for the CT, while the peak arrivals are similar, which reflects both that Li^+^ is adsorbed and that adsorption is noninstantaneous (otherwise the Li^+^ peak arrival would have been retarded).

The redox potential (Figure S3g,h) was quantified as *pe* (computed as *pe* = *EhF*/2.3*RT*, where *Eh* is the measured redox potential, *F* is the Faraday constant, *R* is the gas constant, and *T* is the temperature). Redox potential was measured during sampling, thus representing the mixture of captured waters, which explain the observed fluctuations. The injection of easily degradable DOC led to reduced conditions (i.e., a fast drop in *pe*). A lower *pe* was reached in the ST, where *pe* values decreased from 8 to 3–4 (associated to Mn reducing conditions), whereas in the CT, *pe* dropped to the range of 4–7. Regarding pH, while the pH of the inflow was around 7.5, it was kept between 6.5 and 7 in the whole system. A detailed discussion of the evolution of the different terminal electron acceptors is provided in Section S10 in the SI (Figures S4 and S5). Note that although the acetate injection was equal in the two systems, the response was significantly different. The CT system displayed a larger capacity for DOC and ammonium oxidation as observed in previous works,^30,42^ promoting higher diversity of the redox conditions and, therefore, increased density and diversity of bacterial populations being, all of these, attributed to the presence of the compost reactive barrier (Table S10).

### 3.2 UVFs in Water Samples of the Two SAT Systems

Figure 3a displays the evolution in space (three observation points) and time (cumulative injected water volume) of concentrations of BP-3 and its TPs in water samples. Sampling was scheduled according to the travel times determined in a previous tracer test (S2) so that the same water mass was sampled as it passed through the different sampling points. Similarly, Figure 3b shows the overall mass balance of BP-3 and its TPs of the same sampled water in the inflow and outflow of the two SAT systems.

Overall, UVF concentrations in the sampling points (Figure 3a) did not exhibit a clear trend, which reflects: (1) the inherent variability of UVF concentration in the STE (Table S5), especially at the O-piezometer (this noise is dampened at the downstream observation points due to sorption and dispersion); (2) the complexity of the BP-3 transformation pathway, compromising back and forward reactions conditioned to the redox potential (Figure S2); and (3) the low concentrations, close to the method limit of quantification, especially for TPs. Despite these difficulties, two important issues emerge in Figure 3.

First, CT displayed overall lower concentrations than ST for BP-3 and its TPs in water (Figure 3b). We attribute this to a higher retention and thus degradation capacity of this system induced by the reactive barrier in a similar way as reported for DOC degradation (see Section 3.1). The higher degradation capacity of the CT system can be associated with (1) the geochemical conditions promoted by the reactive barrier facilitated the degradation of UVFs; (2) the CT induced a different microbial population capable of degrading these compounds; and (3) a higher sorption capacity of the system induced by the reactive barrier.

Second, BP-3 and most TPs concentrations dropped in both systems just after the acetate injection, concurring with the tracer peaks, but then rebounded back to levels comparable to the inflow concentration (Figure 3a). This would mean that the presence of acetate enhanced its biodegradation and enlarged the TPs’ production, as reported by Liu et al.^25^ The rebound is explained because: (1) after the acetate peak, the system tended to the prior conditions, especially in ST, and (2) a potential release of BP-3 and TPs from the biofilm phase to the water, promoted by decay of microbial communities and their detachment from the solid surface after the acetate peak, which would favor the dragging of EPS and all sorbed substances.

Regarding TPs formation processes, BP-3 was first biotransformed into BP-1 via demethylation of the methoxy substituent^39^ (Figure 2c). This transformation pathway can occur both in oxic and anoxic conditions.^25^ Note that BP-1 was also present in the influent water (Figure 3, blue shadow zone), meaning it was already present in the inlet water of the WWTP or it was formed there, as observed in Mao et al.^39^ After the injection of LiAc, BP-1 dropped in the ST and remained below the method limit of detection at the CT.

BP-1 biodegradation can lead to the formation of 4DHB.^25^ Similarly and under oxic conditions, BP-3 can form DHMB^46^ (Figure 2c). Both were identified in pore waters and followed a behavior similar to that of BP-3 and BP-1. Prior to the injection, their concentrations were comparable to those in the inflow along the ST and were below the limit of detection in CT. Also, the concentrations of both dropped as the DOC peak reached every observation point and rebounded back to initial operation concentrations after the peak in the ST, but remained comparable to inflow concentrations in A2 and B2 points of the CT.

Finally, 4HB can be formed from 4DHB or, directly, from BP-1 by a hydroxyl group loss^39^ (Figure 2c). Interestingly, this compound was not present in the inflow water. Thus, it was formed in the SAT systems, demonstrating that our systems enhanced the biodegradation of BP-3 regardless of the reactive barrier. Similarly, its concentration was lower in CT, which was completely removed since it was not detected in the outflow (Figure 3b).

Regarding the other monitored UVFs, we did not observe a clear correlation with the acetate injection (S10 in SI). BP-4 was detected in water samples at concentrations between 1 and 2 orders of magnitude higher than the other UVFs, which is consistent with its low degradability both in the WWTP (inflow concentrations were high) and in the SAT systems. AVO, on the other hand, was neither present in the influent water nor in the outflow, but it was detected in water samples from the two SAT systems. As far as we know, there is no literature about the potential formation of AVO as a derivative product of other UVFs. Therefore, we conjecture (1) that AVO reverts back from a conjugate (as a glucuronide conjugate typical from Phase II metabolism) or (2) a potential desorption from the biofilm and sediments, resulting from the dragging of EPS.

### 3.3 Retention of UVFs in Immobile Phase: Biofilm and Sediments

Among the target UVFs, only 4HB, BP-4, and AVO were detected in sediments and biofilm samples. As we did not observe any space correlation for UVFs retained in the solid phases or for retention parameters (bacterial density, EPS, *f*_oc_, or *f*_ow_, Table S10 in the SI), we presented the data in average form before and after the acetate injection (Table 2). Note that the UVFs detected in both biofilm and sediment samples are associated with their accumulation/retention in the immobile phase, that is, interaction with SOM and/or biofilm. As biotraps were previously muffled, the detected UVFs in those samples were only associated with the retention promoted by biofilm (Table 2). Therefore, our results experimentally confirm that biofilm in the porous media is capable of retaining certain UVFs.

**Table 2:** UVFs Concentrations in Biofilm and Sediments Matrices^a^

	retained UVFs in biofilm samples (ng/g)						retained UVFs in sediment samples (ng/g)					
	4HB		BP-4		AVO		4HB		BP-4		AVO	
	ST	CT	ST	CT	ST	CT	ST	CT	ST	CT	ST	CT
initial	ND	ND	ND	ND	10.3 ± 0.03	10.5 ± 0.04	3.70 ± 1.02	0.5 ± 0.9	3.80 ± 6.64	7.3 ± 6.3	3.00 ± 0.03	3.00 ± 0.03
after LiAc	ND	33.1 ± 2.38	32.2 ± 29.5	7.3 ± 6.32	10.5	10.6 ± 0.32	5.3 ± 3.02	15.4 ± 2.69	16.6 ± 1.67	15.4 ± 2.7	3.00 ± 0.03	3.00 ± 0.03

a Concentrations are referred to grams of sampled sediments, where ND means nondetected.

**Figure 4:**
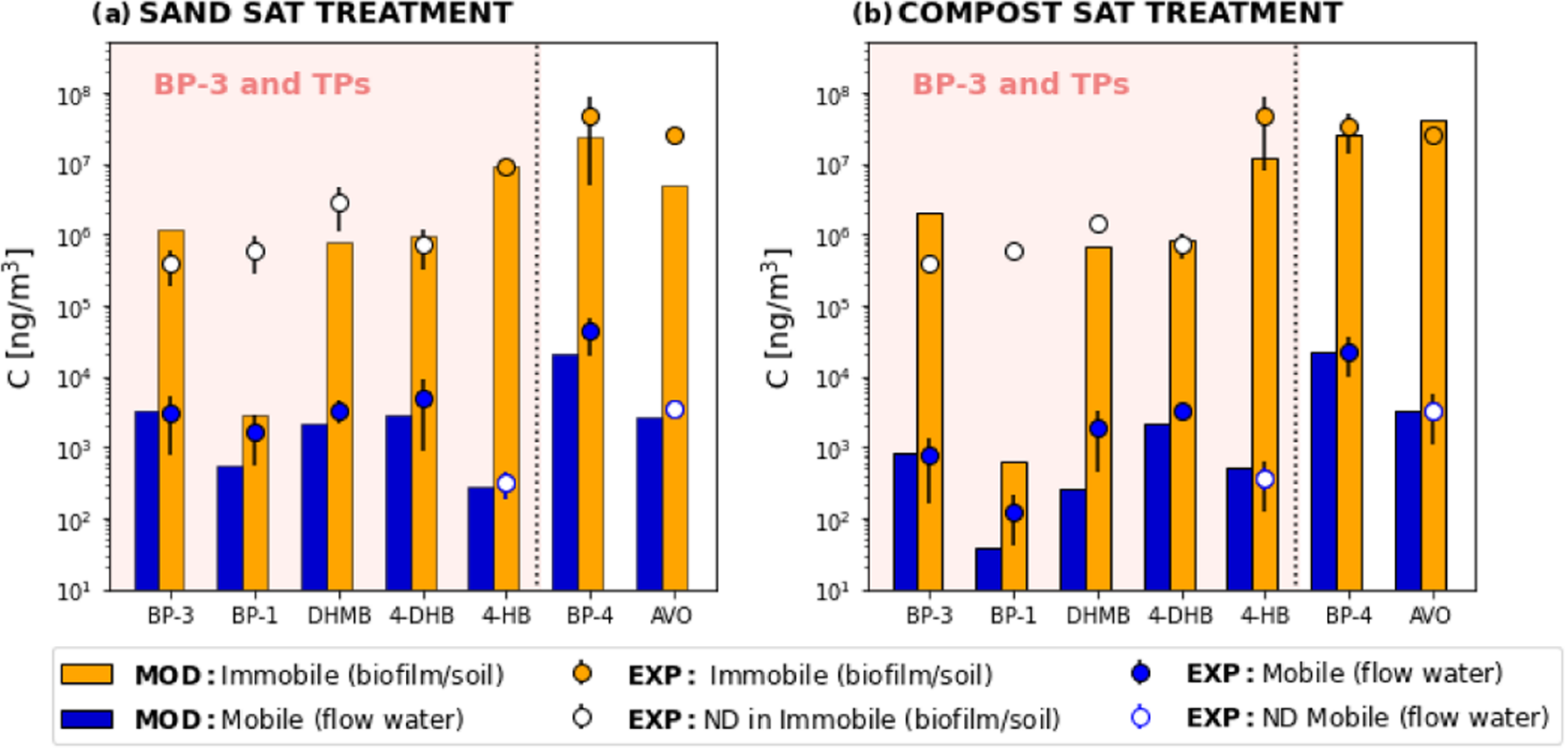
Concentrations in mobile and immobile compartments (expressed in ng per m^3^ of aquifer). Points represent measured concentrations (EXP), whereas bars represent model computations (MOD) in the two compartments. White points refer to undetected compounds (ND) plotted at the detection limit. The salmon represents the BP-3 and TPs compounds.

A further step is to compare the relative importance of mobile and immobile compartments in the fate of UVFs. To do this, we have modeled the concentrations of UVFs in the two compartments using the dual-domain model and compared it with the experimental information (Figure 4), using the biomass concentrations in Table S10. In this analysis, we included all of the UVFs, although we only detected 4HB, BP-4, and AVO. The comparison with the nondetected compounds was done using the detection limit (depicted as white dots in Figure 4). Modeled results for undetected compounds were lower (or very close) to the detection limit (as seen by the white points in Figure 4). This reflects that concentrations are very low and suggests that the current detection limit is still too high for detecting sorbed concentrations in biofilm/sediments.

Two features deserve attention from Figure 4. First, observed (or modeled when not detected) retained UVFs mass in immobile phase is between 1 and 4 orders of magnitude larger than dissolved, with variations depending on the compounds sorption parameters and degradation rates (Figure 4). This accumulation in aquifer sediments was in line with the partition coefficients (*K*_ow_, *K*_oc_, and *K*_d_) of these compounds (Table 1). That is biofilm and sedimentary organic matter present in sediment samples act as real sinks for dissolved UVFs. Second, beyond the affinity of the compounds to the solid phases (values of *K*_ow_, *K*_oc_, and p*K*_a_), the accumulation of TPs also results from the imbalance between its production and degradation. For example, 4HB, which had not been detected in the inflow and had similar solid affinity as BP-3 and the other TPs (see Table 1), displays a high accumulation (its distribution coefficient had to be increased to match observations) and is the last derivative produced along the transformation processes of the BP-3. This shows that the imbalance between its degradation, production, and exchange with the mobile phase is responsible for its retention in biofilm, which is consistent with the findings of Wang et al.^35^ Interestingly, 4HB was not initially present in the biofilm samples and at very low concentration in the sediments ones.

BP-4 was found more frequently and homogeneously in aquifer sediments than in biofilm (Table 2). The presence of BP-4 in water has increased recently because it is substituting BP-3 in some personal care products in order to increase water solubility.^44^ This substitution was motivated by the low affinity of BP-4 for the organic phase (log *K*_ow_ = 0.37) being mainly present in its ionic form (p*K*_a_ = −2.42). Therefore, its retention in sediments is explained by ionic interactions with positive surfaces such as Fe and Mn oxides, which are present in our SAT systems (see S9). Chang et al.^100^ described an interaction between Mn oxides and BP-4. Furthermore, positive surfaces have been described inside biofilms.^35^

AVO was detected in most of the biofilm samples from both systems and at similar concentrations (Table 2). AVO is not a transformation product of BP-3; thus, its accumulation in biofilm is explained by its high affinity to the organic phase in the biofilm (log *K*_ow_ = 4.51) and slow degradability. Note that AVO did not show temporal or spatial variability, showing that it reached an equilibrium concentration that was not dependent on redox conditions or the presence of reactive barrier.

### 3.4 The Biofilm Compartment: Bioaccumulator and Biodegrader in Porous Media

**Figure 5:**
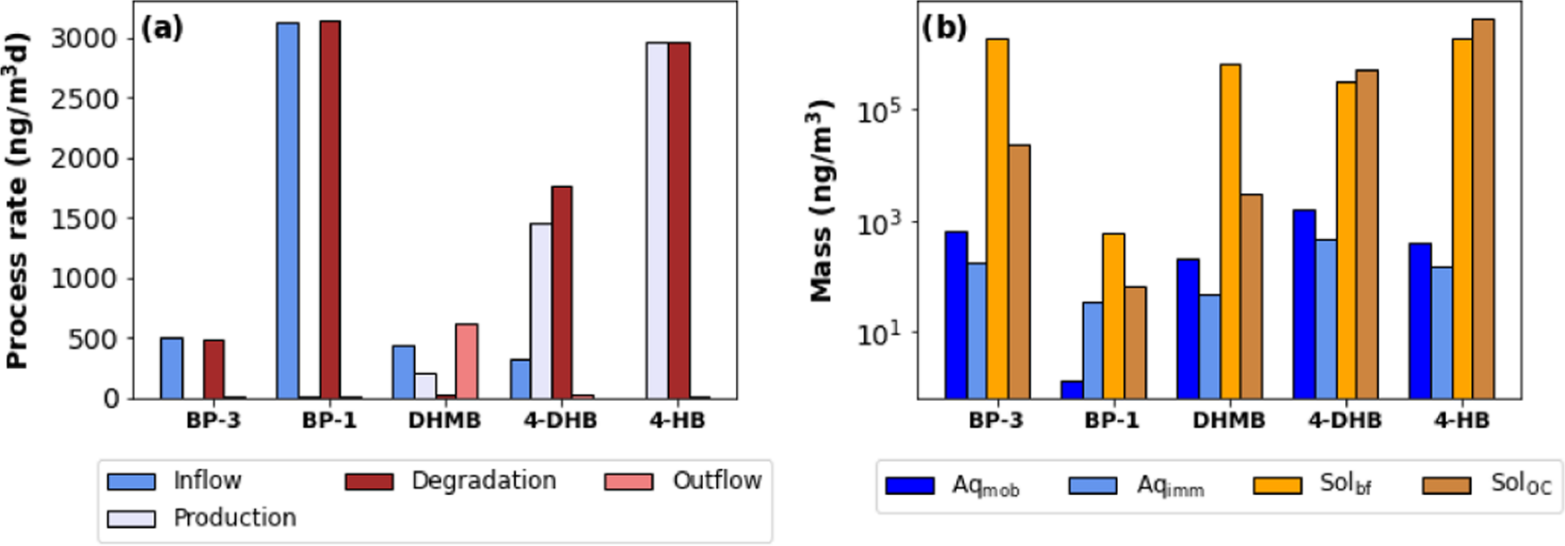
Summary of retention and degradation processes computed for BP-3 and its TPs in the CT. (a) Mass balance terms (ng/m^3^/day) for CT, with cold colors for inputs (inflow and production from parent compounds) and cold colors for outputs (outflow and degradation) and (b) mass retained in the aqueous phase and solid phase (biofilm and aquifer sediments).

In this work, we have detected and quantified, for the first time in porous media, the amount of UVF retained in the nonmobile phases. The developed model reproduced qualitatively the retained mass in the nonmobile phase (Figure 4), in the sense that it reproduces not only the broad range of observed concentrations and yields low concentrations for undetected compounds but also because it simulates the anticipated and observed localization of reactions (4HB being present in the immobile compartment despite its absence in the inflow and outflow). Besides this, it also shows that the compost SAT system presents lower masses of UVFs, despite the fact that its biofilm is more active and SOM content larger. This implies a higher removal capacity. As shown in eqs 1a,b and 2, the degradation rate is effectively multiplied by the retardation coefficient, because so is the residence time. As a result, even compounds that are highly recalcitrant to conventional wastewater treatments may be extensively degraded in biofilms. In short, the suite of observations and model calculations implies that hydrophobic compounds will tend to accumulate in biofilms, which promotes biodegradation even if the degradation rate is very slow (their effective residence time is orders of magnitude larger than that of water). These conclusions are illustrated in Figure 5, which summarizes the mass balance of the various processes controlling the UVF fate of BP-3 and its TPs in the CT (a similar figure for the ST is shown in Figure S5), which underscores the central role of biofilm in the fate of UVFs. This aligns perfectly with the findings in the recent study by Markale et al., where they observed a significant impact of biofilm permeability heterogeneity on biologically driven reactions.^47^

Overall, this study demonstrates that implementing a reactive barrier in an infiltration basin improves the degradation of benzophenone-type UVFs and, especially, of BP-3 and its TPs, and that biofilm acts as an additional environmental compartment favoring the retention and degradation of UVFs in porous media. It does not sway us that these processes can be assumed for other hydrophobic compounds. With this, we want to emphasize that the current understanding of the organic compounds’ fate should incorporate biofilm as a pool capable of bioaccumulating these compounds. Beyond aquifers, the role of biofilms as an additional environmental compartment would imply that aquatic ecosystems would be exposed to a higher dose of UVFs and likely to many other organic pollutants in comparison to those calculated through the measured concentrations in water, suspended particulate matter, sediments, and biota.

## Supporting Information Available

Available detailed site and sampling description; analytical methods; tracer test information; dual-domain model; flow and transport mechanisms; redox geochemistry detailed description; water results for BP-4 and AVO; and aquifer sediments and biofilm parameters (PDF)
