# Supplementary material for: Assessing the Fate of Benzophenone-Type UV Filters and Transformation Products during Soil Aquifer Treatment: The Biofilm Compartment as Bioaccumulator and Biodegrader in Porous Media
Source: Environ Sci Technol. 2024 Mar 11;58(12):5472–82. doi: 10.1021/acs.est.3c08465 (PMC10976883; doi:10.1021/acs.est.3c08465)
Supplement: Supplementary file 1 — es3c08465_si_001.pdf [file es3c08465_si_001.pdf]

Supporting information for

Assessing the fate of Benzophenone-type UV filters and transformation products during Soil Aquifer Treatment: the biofilm compartment as bio-accumulator and bio-degrader in porous media

Sònia Jou-Claus[1,2,3 *], Paula Rodríguez-Escales[1,2], Lurdes Martínez-Landa[1,2], M. Silvia Diaz-Cruz[3], Jesús Carrera[2,3], Adrià Sunyer-Caldú[3,4], Gerard Quintana[3], Cristina Valhondo[2,3]

1 Dept. of Civil and Environmental Engineering. Universitat Politècnica de Catalunya, Jordi Girona 1-3, 08034 Barcelona, Spain

2 Associated Unit: Hydrogeology Group (UPC-CSIC)

3 Institute of Environmental Assessment and Water Research Severo Ochoa Excellence Center, Spanish National Research Council (IDAEA-CSIC), Barcelona 08034, Spain

4 Department of Environmental Science (ACES, Exposure & Effects), Science for Life Laboratory, Stockholm University, Stockholm 106 91, Sweden.

(*) corresponding author: sonia.jou@upc.edu

Summary

S1. Site description and experimental design..................3
S2. Characterization of travel times...............................4
S3. Sampling of different environmental matrices..........5
S5. Standards and reagents..7
S6. Analytical methods...8
S6.1. Hydrochemistry of water..8
S6.2. Total Organic Carbon in solid samples...8
S6.3. Bacterial density and content of polysaccharides in EPS.......................8
S6.4. UVFs analysis in water, aquifer sediment, and biofilm 8
S7. Inflow and outflow UVFs concentrations.................13
S8 Dual domain model 15
S8.1. Equilibrium and mass balance model...15
S8.2. Partitioning 15
S8.3. Equilibrium 16
S9. Flow and transport in the two MAR-SAT Systems..20
S10. Redox geochemistry: understanding the evolution of the different Terminal Electron Acceptors.....................21
S11. Water results for BP-4 and AVO 25
S12. Biofilm characterization and fraction of organic carbon in the two SAT Systems....................................26
S13. Mass balance of BP-3 and TP in ST SAT treatment 27
S13. References...28

S1. Site description and experimental design

The pilot SAT system is in the facilities of the WWTP of Palamós, (Catalonia, Spain) (Figure 1). The site consists of 6 replicates (15 m long, 2.38 m wide, and 1.5 m depth) emulating MAR-SAT systems with an aquifer made up of fine sand (0.1- 0.2 mm grain size) coupled with an infiltration basin of 1.15 m high, 2.38 m wide, and 1.5 m long. The systems are fed with the WWTP secondary effluent, previously homogenized in a reservoir with 24 h retention time. A detailed description of the pilot can be in Valhondo et al., 2020[1].

Two of the six systems were used in this study; one without reactive barrier, made up of sand (sand-treatment, ST), and the other with a 1 m thick reactive barrier made up of sand (49% in volume), vegetal compost (49%) and clay (2%) (compost-treatment, CT) installed at the bottom of the infiltration basin. The systems are equipped with monitoring points distributed along the flow path (10 piezometers and the inflow and the outflow points of the systems) (Figure 1). Nine piezometers were installed in three sections, at 1.3 m (Section A), 6.3 m (section B), and 12.3 m (Section C) from the infiltration basin. Every section contains three PVC piezometers of 53 mm diameter, each open (10 cm screened interval) at a different height from the aquifer base: 0.1-0.2 m (piezometer 1), 0.6-0.7 m (piezometer 2) and 1.2-1.3 m (piezometer 3), referred hereafter as Si piezometer; S=A, B, C, indicates the section where the piezometer is placed and i=1,3 indicates the depth of the screening interval. Additionally, a fully screened inclined piezometer at the base of the reactive barrier can collect water exiting the barrier, named oblique and referred hereafter as "O". Finally, outflow is integrated and collected by means of a discharge pipe installed at the base of the aquifer end. Head measured as pressure, electrical conductivity (EC), and temperature were measured regularly with CTD/CD-Divers (Schlumberger water services, Delf, The Netherlands).

The experiment consisted of injecting 57 L of water from the WWTP spiked with lithium acetate (LiAc) at 17.8 mg/L in the infiltration basins of the two selected systems ST-, and CT- and the subsequent monitoring of the hydrochemical parameters and UVFs concentrations in water, and solid samples.

S2. Characterization of travel times

Electrical conductivity of the WWTP effluent was used to obtain the breakthrough curves (BTCs) at the different sampling points. Figure S.1 shows the BTCs at different monitoring points and the sampling periods that were selected to capture the BTCs at each piezometer.

SAND TREATMENT

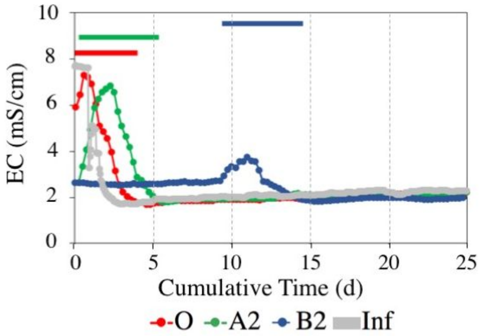

MONITORING POINT	MONITORING TIME (d)
Oblique-ST	0.5; 0.6; 0.7; 0.83; 0.95; 1; 1.52; 1.76; 1.90
A2-ST	1.79;2.55;2.76;3.54;4.71;5.53;6.67
B2-ST	6.97;9.69;11.91;13.94;16.60;18.77;20.69;23.66
Soil	0; 6; 8; 28; 61
Biofilm	0; 6; 8; 28; 61

COMPOST TREATMENT

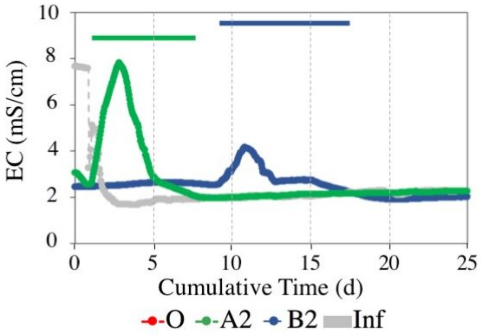

MONITORING POINT	MONITORING TIME (d)
Oblique-CT	0.52;0.61;0.70;0.85;0.96;1.01;1.52;1.80;1.91
A2-CT	1.82;2.56;2.74;3.52;4.73;5.54;6.68
B2-CT	6.98;9.72;11.93;13.95;16.61;18.78;20.70;23.67
Soil	0; 6; 8; 28; 61
Biofilm	0; 6; 8; 28; 61

Figure S1. Evolution of Electrical conductivity (EC) peak *versus* cumulative time in infiltration water (Inf) and observation points (O, A2, and B2) of the ST and CT systems measured before the experiment with the systems operated under the same conditions that during the experiment. The horizontal straight color bars represent the monitoring intervals of each sampling point in this experiment, scheduled based on the EC data.

S3. Sampling of different environmental matrices

50 **samples of water** were collected the inlet (INF: recharge water), piezometers and outlet of the two SAT systems. Samples were collected after purging the piezometers using drive pumps. Physicochemical field parameters (EC, pH, redox potential (Eh), and temperature) were measured *in situ* with a multiparameter probe (YSI Inc. Yellow Springs, OH, USA). Alkalinity was measured in the field with an alkalinity test kit (Merck Millipore, Darmstadt, Germany).

Water hydrochemistry characterization consisted in the analysis of dissolved organic carbon (DOC), NH_4^+, major cations and anions. The water samples for DOC and ions determinations were filtered in the field through 0.22 μm nylon membrane filters. Aliquots for DOC were collected in muffled glass bottles of 20 mL and acidified with HCl, samples for major cations and anions were collected in polypropylene bottles of 20 and 40 mL, respectively and those for cations determination were acidified with HNO_3. The samples were shipped to laboratory under cool conditions and stored under 4 °C. Samples for UVFs analysis were collected in amber glass bottles of 150 ml, immediately frozen, and kept in the dark to prevent from photo- and bio-degradation.

Aquifer sediment samples to analyze organic carbon were stored in 13 mL polypropylene bottles (5 g per sample) and kept under 4°C until analysis. Aquifer sediment samples to analyze UVFs were stored in muffled amber glass bottles (20 g per sample) and kept in the dark at -20°C until analysis.

Bio-traps samples for EPS analyses were collected in 13 mL polypropylene bottles (7g per sample) and were stored frozen until analysis. Samples for bacterial analyses

were collected in muffled glass bottles (2.3 g per sample) where 10 mL of filtered water (0.2 μm filter pore) from the site and 100 μL of formaldehyde (37%) were added. They were stored under 4 °C until analyses. Samples for UVFs analyses in biofilm (200-400 g per sample) were collected in 200 mL muffled amber glass bottles and stored frozen until analysis.

S5. Standards and reagents

All the UVFs standards used were of >98% of purity. BP-3, BP-4, AVO, BP-1 and 4-DHB were purchased from Sigma Aldrich (Darmstadt, Germany). DHMB was obtained from Merck (Darmstadt, Germany). The isotopically labelled internal standards, 2-hydroxy-4-methoxy- 2′,3′,4′,5′,6′-d5 (BP3-d5), (±)-3-(4-methylbenzylidene-d4) camphor (4MBC-d4), and 5-(2,5-dimethylphenoxy)-2,2-bis(tri-deuteriomethyl) pentanoic acid (GMF-d6) were from CDN isotopes (Quebec, Canada).

LiAc was 99% of purity and was purchased from Thermo Fisher Scientific (Waltham, MA, USA). HCl, HNO_3 and CH_2O analytical grade were provided by Merck (Darmstadt, Germany).

S6. Analytical methods

S6.1. Hydrochemistry of water

DOC was measured with a TOC-VCSH analyzer Shimadzu (Kyoto, Japan) in the filtered water samples. Cationic species were analyzed by inductively-coupled plasma optical emission spectroscopy (ICP- OES) using an iCAP 6500 instrument (Thermo Fisher Scientific). NH_4^+ was analyzed using an Ion Selective Electrode (Thermo Fisher Scientific, Massachusetts, USA). Major anions (F^-, Cl^-, NO_3^-, Br^-, NO_2^-, PO_4^{3-} and SO_4^{-2}) were determined by ion chromatography (Dionex, Sunnyvale, CA, USA) using an Ionpack AS9 2x250 mm column and Na_2CO_3 9 mM solution as mobile phase.

S6.2. Total Organic Carbon in solid samples

TOC was determined by organic elemental analysis (Thermo Scientific, Milan, Italy). Before the analysis the samples were dried in an oven at 50°C for 24 h and then were ground with a ball mill. Mineral carbonate from the samples was removed with 6M HCl to isolate organic carbon. Subsequently, 20 mg of each sample were weighed and V_2O_5 was added to facilitate combustion. The samples were analyzed at 120 mL/min flow, combustion oven at 1000 °C, chromatographic column at 60°C, and oxygen loop of 10 mL at 100 kPa.

S6.3. Bacterial density and content of polysaccharides in EPS

Bacterial density and EPS determinations are described in Perujo et al.[2]

Bacterial density was measured by flow cytometry and results are reported as bacterial cells $\cdot 10^7$/g sediment dry weight (dw). EPS were extracted by a cation exchange resin and the content of polysaccharides was measured spectrophotometrically.

S6.4. UVFs analysis in water, aquifer sediment, and biofilm

Water samples for UVFs analysis were vacuum filtered twice, first using 1 mm glass fiber filters (Whatman, Fairfield, CT, USA) and then through 0.45 μm nylon

membrane filters and stored at -20 °C in the dark until analysis. Analytical determinations were performed using on-line solid phase extraction coupled to high performance liquid chromatography-tandem mass spectrometry (on-line-SPE-HPLC-MS/MS), as described in Gago-Ferrero et al.[3]. Briefly, 5 ml of the water samples were extracted and purified in an automated on-line SPE-LC Symbiosis™ Pico (Spark Holland. Emmen, The Netherlands) instrument using PLRPs on-line SPE cartridges. The trapped analytes were eluted from the SPE cartridge with the chromatographic mobile phase and introduced into an LC analytical column Hibar Purosher® STAR® HR R-18 (50 mm × 2.0 mm, 5 µm). The mobile phase consisted of HPLC-grade water and acetonitrile (ACN) with 0.1% formic acid as a modifier to analyze in positive ionization mode, and 5 mM of NH_4Ac as modifier to when analyzing in negative ionization mode. MS/MS detection was performed in a 4000 QTRAP™ MS/MS mass spectrometer (Applied Biosystems-Sciex. Foster City, USA) under selected reaction monitoring mode (SRM) for improved selectivity and sensitivity. Analyses were run in both positive and negative modes using an electrospray ionization source (ESI+, ESI). Quantification was performed by isotopic dilution using isotopically labelled internal standards specified in the chemicals section. Method limits of detection (LODs) and quantification (LOQs) are presented in Table S1.

Table S1. LODs and LOQs for water analysis.

	BP3	BP1	4HB	4DHB	DHMB	AVO	BP4
LOD (ng/L)	0.20	0.16	0.37	0.22	0.21	0.50	0.46
LOQ (ng/L)	0.65	0.53	1.23	0.75	0.71	1.66	1.52
R^2	0.9995	0.9997	0.9982	0.999	0.9994	0.9967	0.9973

Aquifer sediment and biofilm samples were analyzed using a QuEChERS-based methodology originally developed for the determination of UVFs in vegetables[4]. In

the case of the biofilms, the development and further validation of the analytical method was performed.

Sampling and sample extraction for aquifer sediment and biofilm

Bags with sterilized sand were installed in 3 piezometers (O, A2, and B2) in a pilot sediment aquifer treatment (SAT) system (Figure 1). After a month, during which biofilms were expected to grow on the bags as water flowed through the systems, the bags were extracted, opened, and the sand inside them was collected with a spatula (c.a. 300 g per sample) and transferred to 200 mL muffled amber glass bottles.

Biofilm samples were lyophilized. Commercial QuEChERS kits from BEKOlut® (citrate and PSA-Kit-02 kits) were used for extraction and purification of 1 g dw of biofilm plus 5 g dw of sediment. Finally, an aliquot of 5 mL from the extract was evaporated and reconstituted to 1 mL with MeOH.

Instrumental Analysis

Separation and quantification of the target analytes were performed by high-performance liquid chromatography in a Hibar Purosher® STAR® HR R-18 (50 mm × 2.0 mm, 5 μm) column using a Symbiosis™ Pico instrument from Spark Holland (Emmen, The Netherlands) attached to a 4000 QTRAP mass spectrometer from Applied Biosystems-Sciex (Foster City, USA). All sample extracts were analyzed in two ionization modes; positive ionization and negative, using electrospray ionization (ESI+ and ESI-). A binary gradient with a flow rate of 0.3 mL/min was used. For ESI+, the mobile phase A contained 0.1 % formic acid in water, while mobile phase B was MeOH. Regarding ESI-, 5 mM ammonium acetate in water was employed (A), while MeOH was used as the organic phase (B). The elution program in positive mode started with 5% MeOH, which was increased to 75% during 6.8 min, to finally reach 100% and held for 5 min before returning to initial conditions during 2 min (3 min of equilibration). In ESI- the elution was programmed as follows: initial

conditions were set at 5% MeOH, which was increased to 50% during 3 min, then set at 90% during 3 min, before reaching 100% during 7 min. Initial conditions were reached after 5 min and equilibrated for 2 min. The sample injection volume was set at 20 µL. The detector was operated in selected reaction monitoring (SRM) mode. Two SRM transitions were recorded, the most intense for quantification, and the other, for identity confirmation. All data were acquired and processed using Analyst v. 1.4.2 (Applied Biosystems).

Quantification was performed using calibration curves and the addition of the isotopically labeled internal standards specified in the chemicals section. LC-MS/MS parameters applied are listed in Table S2.

Table S2. Retention time (t_R), SRM transitions, internal standard (IS), and ionization parameters for each compound.

	BP1	BP3	BP4	4HB	4DHB	DHMB	AVO
**t_R (min.)**	9.64	11.11	7.61	8.95	7.74	9.99	13.67
**Precursor (m/z)**	215	229	307	199	215	245	311
**1st transition product (m/z)**	137	151.04	211	121	121	121	161
**CE (eV)**	20.1	21.6	39	18.6	20.1	19.1	24.6
**2nd transition product (m/z)**	81	95	227	105	93	151	135
**CE (eV)**	33.1	32.6	24	18.1	29.6	20.6	23.1
**RF Lens (V)**	46	68	95	49	53	58	59
**IS**	BP3-d5	BP3-d5	BePB-d4	BP3-d5	BP3-d5	BP3-d5	BP3-d5

Quality assurance and quality control

For QA/QC, quality control standards were interspersed between the samples to ensure the accuracy of the analyses. Blank analyses were performed with HPLC water. The calibration curves were built through mix standard solutions (in MeOH) at 1, 3, 5, 10, 30, 50, 100, 300, 500, and 700 ng/mL.

Method validation

Analytical calibration curves were constructed according to the individual response range of each analyte. In order to validate the method for the biofilm, MLODs and method limits of quantification (MLOQs) were determined as three and ten times, respectively, the standard deviation of the calibration curve divided by the slope. Accuracy was evaluated by the recovery rates of each standard spiked in the biofilm (a representative mixture of the biofilms), determined in four replicate spiked extracts at two concentration levels, 5 and 100 ng/mL of the mix standards solutions, and measured 3 times (n=9). The precision was expressed as a percentage of relative standard deviation (RSD (%)) for each concentration level.

The recovery rates achieved ranged from 73.2 % to 138% (except for 4HB, which presented too low recoveries at the high concentration level). The obtained recoveries are listed in Table S3. The lowest recovery corresponded to 4HB, and the highest for DHMB.

The MLODs, MLOQs, and RSDs are listed in Table S4, along with the determination coefficient for each compound. The MLODs for biofilm ranged between 0.18 and 0.87 ng/g dw, and the MLOQs ranged between 0.60 and 2.89 ng/g dw, showing that the method presents good sensitivity. The RSD values were below or equal to 20%, indicating good precision.

Table S3. Recoveries of selected CECs from biofilm at two spike levels. Four replicates for each level.

	BP3	BP1	BP4(-)	4DHB	DHMB	AVO
**5 ng/g dw (1)**	129.4	93.6	113.2	81.6	125.2	116.0
**5 ng/g dw (2)**	103.0	81.6	115.8	88.2	131.2	95.2
**5 ng/g dw (3)**	124.0	73.2	104.4	92.4	128.6	109.4
**5 ng/g dw (4)**	107.2	119.2	72.5	97.2	127.2	90.4

**100 ng/g dw (1)**	86.4	96.3	73.4	122.8	122.1	107.6
**100 ng/g dw (2)**	79.6	99.3	104.2	113.7	138.0	84.4
**100 ng/g dw (3)**	86.5	79.5	94.0	94.2	117.0	85.6
**100 ng/g dw (4)**	88.0	94.2	65.2	116.3	121.9	85.2

Table S4. Limits of detection (MLODs) and quantification (MLOQs) of the method and coefficient of determination (r2) for each compound analyzed in the method. (-) analysed unde ESI- ionization mode, Relative standard deviation (RSD) in %. dw, dry weight.

	BP3	BP1	BP4(-)	4HB	4DHB	DHMB	AVO
**MLOD (ng/g dw)**	0.2	0.8	0.9	0.8	0.4	0.9	0.6
**MLOQ (ng/g dw)**	0.6	2.8	2.8	2.7	1.2	2.	1.9
**r2**	0.9996	0.9912	0.9907	0.9917	0.9984	0.9902	0.9958
**RSD at 5 ng/g**	13	20	20	2	6.6	2.5	12
**RSD at 100 ng/g**	3.8	8.8	18	0.2	1	9.1	11

Besides being fast and simple, the method presents good precision, recoveries, and limits of detection and quantification, demonstrating the methodology's suitability for quantificatifying UVFs in biofilms.

S7. Inflow and outflow UVFs concentrations

Table S5. UVFs concentration at the inflow of the SAT systems, where time 0 represents the moment of the injection of LiAc and N.D. represents non-detected compound.

Time<br>d	BP-3<br>ng/L	BP-1<br>ng/L	BP-4<br>ng/L	4-HB<br>ng/L	4-DHB<br>ng/L	DHMB<br>ng/L	AVO<br>ng/L
0.00	104	348	1590	n.d.	12.9	54.3	n.d.
0.51	31	316	712	n.d.	17.8	30.3	n.d.
0.86	21.20	116	1560	n.d.	9.87	7.1	n.d.
1.03	43.90	230	943	n.d.	7.21	4.29	n.d.
1.63	13.60	101	1150	n.d.	9.24	14.4	n.d.

1.83	19.70	151	1300	n.d.	8.49	12.1	n.d.
2.62	13.20	87.7	1050	n.d.	14.3	8.98	n.d.
3.01	15.90	247	1090	n.d.	87.8	35.1	n.d.
3.51	14.20	128	1090	n.d.	17	15.3	n.d.
4.75	1.26	5.1	350	n.d.	22.3	10.7	n.d.
5.56	22.80	137	820	n.d.	3.66	24.2	n.d.
6.69	13.80	11.4	1120	n.d.	7.88	9.94	n.d.
7.56	28	175	558	n.d.	3.01	56.1	n.d.
9.74	15.50	62.6	889	n.d.	17.6	20.8	n.d.
11.19	17.40	253	793	n.d.	10.5	29	n.d.

Table S6. UVFs concentration at the outflow of the ST-SAT system, where time 0 represents the moment of the injection of LiAc and n.d. represents non-detected compound.

Time<br>d	BP-3<br>ng/L	BP-1<br>ng/L	BP-4<br>ng/L	4-HB<br>ng/L	4-DHB<br>ng/L	DHMB<br>ng/L	AVO<br>ng/L
14.00	9.9	n.d.	484	0.37	n.d.	n.d.	n.d.
16.78	14.5	n.d.	336.5	0.37	n.d.	n.d.	n.d.
17.67	19.1	n.d.	189	0.37	n.d.	n.d.	n.d.
20.48	n.d.	3.61	n.d.	10.5	4.56	98.5	n.d.
24.53	n.d.	n.d.	835	0.37	4.86	97.1	n.d.
26.72	n.d.	n.d.	835	0.37	4.86	97.1	n.d.

Table S7. UVFs concentration at the outflow of the CT-SAT system, where time 0 represents the moment of the injection of LiAc and n.d. represents non-detected compound.

Time<br>d	BP-3<br>ng/L	BP-1<br>ng/L	BP-4<br>ng/L	4-HB<br>ng/L	4-DHB<br>ng/L	DHMB<br>ng/L	AVO<br>ng/L
14.00	9.9	n.d.	484	n.d.	n.d.	n.d.	n.d.
17.67	n.d.	n.d.	212	n.d.	n.d.	n.d.	n.d.
20.48	n.d.	n.d.	483	n.d.	n.d.	12.2	n.d.
24.53	n.d.	n.d.	n.d.	n.d.	n.d.	40.9	n.d.
26.72	n.d.	n.d.	835	n.d.	4.86	97.1	n.d.

S8 Dual domain model

S8.1. Equilibrium and mass balance model

Equilibrium and mass balance equations are derived by accounting for partitioning and degradation processes in a two-compartment model (Figure 2). The immobile water compartment represents water in biofilms, solid organic matter, and isolated pores. Therefore, sorption (both absorption into organic solids and adsorption onto mineral surfaces and charged organic matter) occurs primarily in the immobile compartment. It is also assumed that microbial communities responsible of degradation reactions live in biofilms. They are assumed mature, so that degradation is limited by the concentration of the compound and can be taken as first order. Instead, the mobile compartment represents free flowing water, so that sorption and degradation are neglected. These compartments are characterized by their ϕ_{im} and ϕ_{im} $[\mathrm{V_{imw}V_{aq}^{-1}}]$ (notice that, for clarity, we choose to indicate not only the units but also what they apply to, so that $\mathrm{V_{imw}}$ is the volume of immobile water, and we use roman text to specify units, reserving italics for variables). Since mobile and immobile porosity are referred to the total volume of the medium, the total porosity is $\phi = \phi_m + \phi_{im}$.

S8.2. Partitioning

We assume the UVFs to distribute among four phases: water, biofilm, the mineral (surfaces) and the organic aquifer sediment fractions. Thermodynamic equilibrium is controlled by distribution coefficients, which relate the sorbed and dissolved UVFs masses: $S_{p,i} = K_{p,i}c_i/\rho_w$, where $S_{p,i}$ $[\mathrm{M_iM_p^{-1}}]$is the mass of species i retained by the p phase, $K_{p,i}$ $[\mathrm{M_wM_p^{-1}}]$ is the water-p (also termed partition) coefficient, and we assume that that water concentration c_i is expressed as mass of solute per unit volume of water. The total retained mass per unit volume of sediments, m_i $[\mathrm{M_iV_{\mathit{sed}}^{-1}}]$, is the sum of all of them $m_i = \sum_p \rho_p K_{p,i}c_i/\rho_w$, where ρ_p $[\mathrm{M_iV_p^{-1}}]$ is the mass of phase p per unit

volume of medium. The medium is usually adopted as the whole sediment mass. However, here, we adopt the immobile compartment. Therefore $\rho_{pim} = f_p \rho_{im}$, where f_p $\left[\mathrm{M_p M_{im}^{-1}}\right]$ is the mass fraction of phase p in the immobile compartment mass, and ρ_{im} $\left[\mathrm{M_{im} V_{im}^{-1}}\right]$ is the bulk density of the immobile compartment. Note that $\rho_p = F_{im}\rho_{pim}$, where F_{im} $\left[\mathrm{V_{pm} V_{im}^{-1}}\right]$ is the volumetric fraction of the immobile compartment. Since we are assuming that sorbing phases are part of the immobile compartment, the total sorption capacity $\sum_p \rho_p K_{p,i}$ is independent of whether a single or a multiple compartment model is adopted. As for the value of partitioning coefficients, we used the K_{ow} and K_{OC} of Table 1 of manuscript. The ionic adsorption coefficient, K_d, was set to zero for compounds with pKa outside the 7-8 interval, and calibrated to a constant value of 2 for the rest in the ST and scaled with f_{ow} for the CT. As it turned out, the K_d of 4HB had to be dramatically increased to reproduce observations. Therefore, the usual computation of retention capacity remains valid. For mass balance computations, it is convenient to work with retardation coefficients, which relate the total mass in the medium (both is solution and sorbed) to that in solution.

$$R_{im,i} = 1 + \frac{\sum_p \rho_p K_{p,i}}{\phi_{im}\rho_w} \qquad \text{(A1)}$$

S8.3. Equilibrium

Equilibrium is reached after transport over long term. That is, we need to, first, write the mass balance in the immobile compartment of Figure 2, and then, derive the immobile concentration, c_j^{im}, assuming known the mobile concentration, c_j. We assume, for simplicity that all compounds transfer between mobile and immobile zones at the same rate, $\alpha\,[T^{-1}]$, inverse of the mean residence time of inert compounds in the immobile zone($t_{Rim} = 1/\alpha$). This value was fixed to 50 1/d, which corresponds to a pore molecular diffusion of 0.6 10-9 m^2/s into a 1mm thick domain.

That is, the mass of compound j transferred per unit volume of immobile water and unit time is $\alpha(c_j - c_j^{im})$. Mass balance in the immobile zone, per unit volume of immobile water (note that all terms are those of Figure 2, but divided by $\phi_{im}V$) is:

$$R_{im,j}\frac{dc_j^{im}}{dt} = \alpha(c_j - c_j^{im}) + \sum_{p=1}^{N_{pj}} \lambda_{p,j}R_{im,p}c_p^{im} - R_{im,j}\sum_{d=1}^{N_{dj}} \lambda_{j,d}c_j^{im} \tag{A2}$$

where N_{pj} is the number of parents of the j-th compound (for example 4-HB has two parent compounds, BP1 and 4-DHB) with degradation rates, $\lambda_{p,j}$, N_{dj} is the number of daughters of the $j-th$ compound (for example BP-3 has two daughters, BP1 and DHMB). These degradation rates were calibrated manually. In addition to the degradation chain of Figure 2, some additional degradation to unknown TPs had to be assumed for some of the compounds for a fair calibration (values displayed in Tables S6 and S7).

Equation A1 illustrates several relevant features. First. The mean residence time can be easily obtained by setting all λs to zero and integrating, which yields, $t_{Rim,j} = R_{im,j}/\alpha$. This can be very large for compounds with large $R_{im,j}$, which implies that degradation must be accounted for. Second, the degradation term is multiplied by the retardation factor, so that it may affect a significant fraction of the compound. In fact, it is convenient to define $\lambda_{i,j}^R = \lambda R_i$, and define the total degradation rate, $\lambda_{j,T}^R = \sum_{d=1}^{N_{dj}} \lambda_{j,d}^R$. Equilibrium is obtained by setting the time derivative to zero, which yields to:

$$c_j^{im} = \frac{\alpha c_j + \sum_{p=1}^{N_{pj}} \lambda_{p,j}^R c_p^{im}}{\alpha + \lambda_{j,T}^R} \tag{A3}$$

The total (in solution plus sorbed) concentration is $c_{tot,j}^{im} = R_{im,j}c_j^{im}$. But this is inconvenient, as it represents the mass per unit volume of immobile water. For

comparing with measurements, it is best to express the mass per unit volume of sediments.

$$S_j^{im} = \phi_{im}(R_{im,j} - 1)c_j^{im} \tag{A4}$$

These equations quantify that equilibrium immobile concentrations results from two sources: transfer from (to) the mobile region, and production from parent compounds and/or biodegradation. Note that little is gained by adopting the two compartments model for non-degrading compounds because, if all λs are zero, then $S_j^{im} = R_{im,j}c_j$, which is identical to the traditional model.

The above expression relates mobile and immobile (including sorbed) concentrations. But, to relate them with inflow concentrations, we need to solve also the mass balance in the mobile zone. Since we are assuming that all reactions take place in the immobile zone, the mass balance includes only inflow, outflow and exchange, which reads (expressed per unit volume of medium, i.e., dividing by V the terms in Figure 2)

$$\phi_m \frac{dc_j}{dt} = \frac{q}{L}\left(c_j^{Inp} - c_j\right) - \alpha\phi_{im}\left(c_j - c_j^{im}\right) \tag{A4}$$

where A and L are the mean cross-sectional area and length, respectively, and $q = Q/A$ is the mean flux. The steady-state solution of this problem is

$$c_j = \theta c_j^{Inp} + (1-\theta)c_j^{im} \tag{A5}$$

Where $\theta = {}^{q}/(q + L\alpha\phi_{im})$, which can be seen as the ratio flow-through to total (flow-through plus exchange along the whole medium) fluxes.

$$c_j = \frac{\theta\left(\alpha + \lambda_{j,T}^R\right)}{\left(\theta\alpha + \lambda_{j,T}^R\right)} c_j^{Inp} + \frac{(1-\theta)}{\left(\theta\alpha + \lambda_{j,T}^R\right)} \sum_{p=1}^{N_{pj}} \lambda_{p,j}^R c_p^{im} \tag{A6}$$

The model parameters are shown in Tables S7 and S9. Flow and transport parameters were fitted with the experimental result and were according to previous experimental tracer tests determined in the Palamós site (Aguirre et al. 2022). Degradation parameters were fitted by hand with experimental data. These values were not able to be compared with the literature since non information was found.

Table S8. General parameters adopted for the dual domain model

Parameter	ST	CT
$L\ (m)$	15	
$A\ (m^2)$	3.525	
$Q\ (m^3/d)$	1.05	
$\alpha(1/d)$	50	
ϕ_{TOT}	0.25	
ϕ_{im}	0.1	0.2
ϕ_m	0.15	0.05
f_{OC}	0.00098	0.0014
f_{OW}	0.0134	0.0929

Table S9. Degradation and sorption parameters adopted for the dual domain model

Parameter	BP-3	BP-1	BP-4	4-HB	4-DHB	DHMB	AVO
$K_{OC,j}$	1259	2884	91.2	1738	2818	2089	1698
$K_{ow,j}$	6166	912	2.34	1047	155	6607	32359
$K_{d,j,ST}$	2	2	1.5	4000	2	0	0
$R_{im,j,ST}$	880	191	31	78000	89	908	4361
$\lambda_{p,d}$(1/d)	BP1, 2.4E-5<br>DHMB, 2.4E-4	4HB, 1.6E+1<br>4DHB, 5.0E+0			4HB, 5.0E-2		
$\lambda_{p,unk}$(1/d)	3.0E-4	1.0E+0	4.0E-3	3.0E-4		7.1E-7	1.0E-2

S9. Flow and transport in the two MAR-SAT Systems

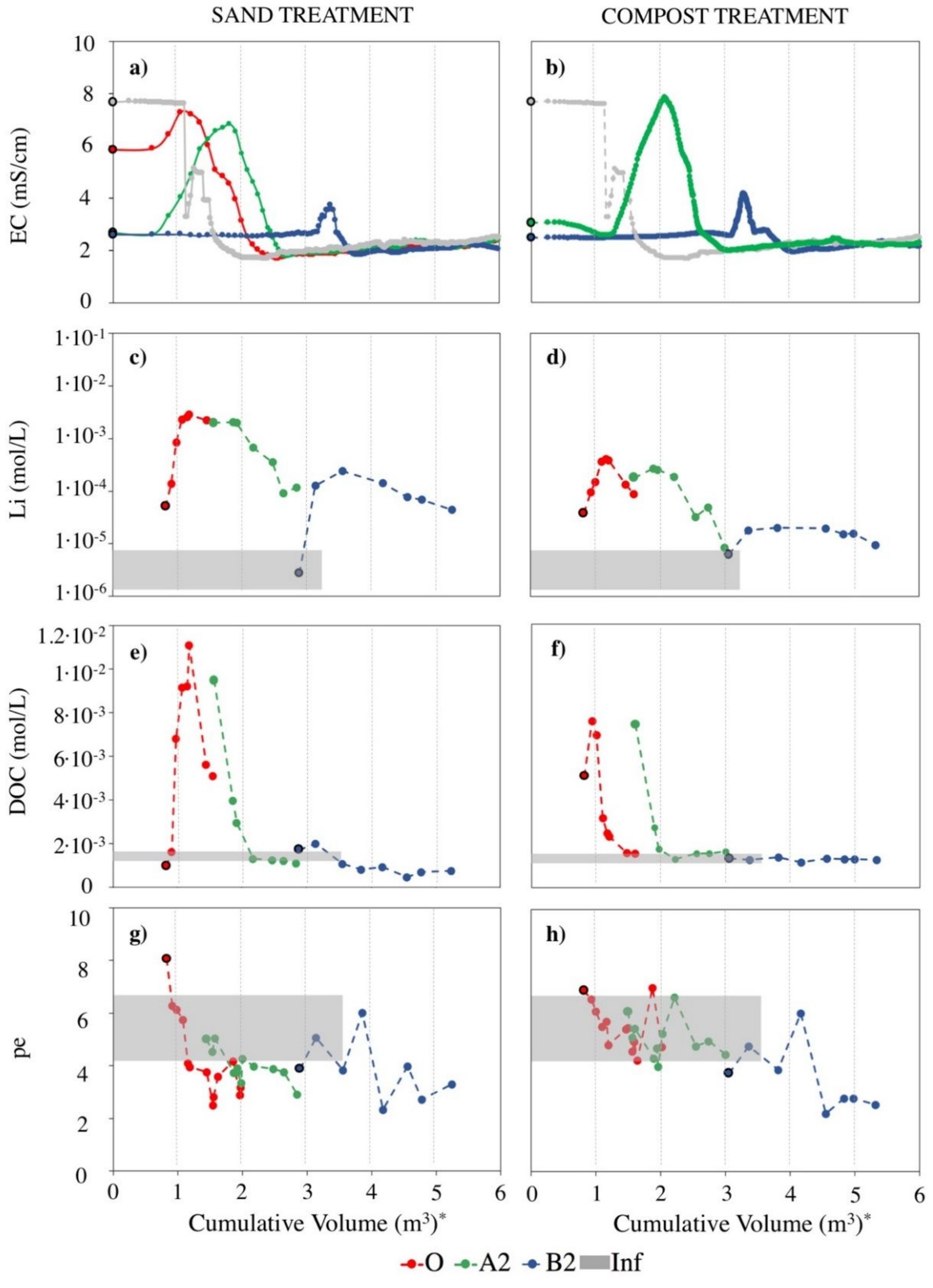

Figure S2. Evolution of electrical conductivity (a and b), redox potential pe (g and h), concentrations of lithium (c and d), and dissolved organic carbon (DOC) (e and f)), the square root of cumulative volume along the two SAT systems. Electrical conductivity in the observation point O of the CT was not acquired. The shadow rectangle represents the variability of concentration/value in the influent. The dots with the black border represent the conditions before the injection of lithium acetate.

S10. Redox geochemistry: understanding the evolution of the different Terminal Electron Acceptors

The redox potential (Figure 2f and 2g) was quantified as pe (computed as $pe = EhF/2.3RT$, where Eh is the measured redox potential, F the Faraday constant, R the gas constant, and T temperature). Redox potential was measured during sampling, thus representing the mixture of captured waters, which explain the observed fluctuations. The injection of easily degradable DOC leads to reducing conditions (i.e., a fast drop in pe). A lower pe was reached in the ST, where pe values decreased fast, from 8 to 3 - 4 (associated to Mn reducing conditions) than in the CT, where pe dropped to the range of 4-7. This latter range is usually associated to denitrification processes, but nitrate was virtually absent in the inflow which is ammonium rich. These differences in the redox potential may be explained by higher denitrification rates in the CT (Figure 3). Under normal operation (first point in the graphs of all observation points), ammonium depletion occurred in both systems, but much more in the CT, whose NH_4^+ dropped some 4 mmol/L, than in the ST. The fact that NO_3^- only increased 0.1 mmol/L implies that denitrification was also occurring in the CT. The injection of acetate leads to reducing conditions, which might explain the slight decrease in ammonium (nitrification is hindered) in the ST and the drop in nitrate (denitrification is also hindered), but not the drop of ammonium in the CT. At this point, we conjecture the potential existence of other ammonium oxidation processes leaded by the presence of other electron acceptors such as manganese[5], iron[6], or even the presence of annamox [7].

Nevertheless, we do not have the enough experimental evidence to confirm any of these processes. Note that, downstream of the CT barrier, NH_4^+ builds up. This can be explained by the potential desorption of sorbed ammonia until reach equilibrium conditions.

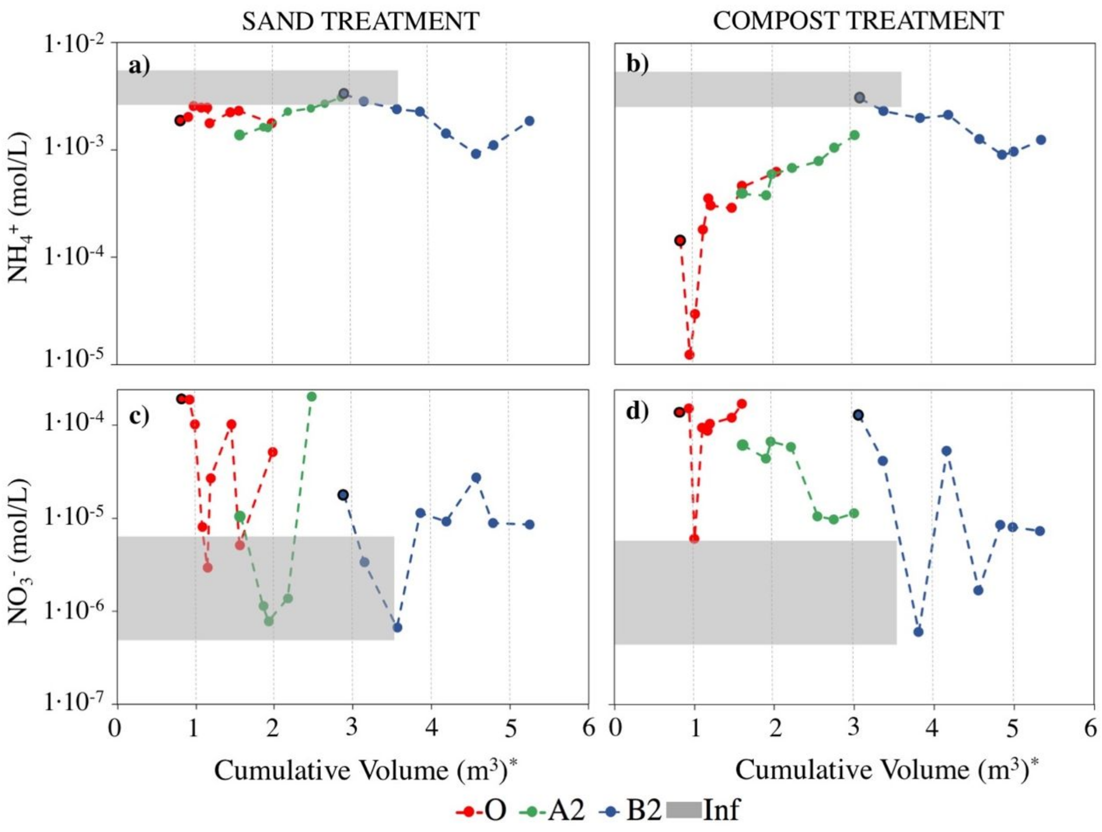

Figure S3. Evolution of the concentration of ammonium (a, b) and nitrate (c, d) *versus* square root of the cumulative volume along the two SAT systems. The shadow rectangle represents the variability of concentration/value in the influent. The dots with black border represent the conditions before the injection of lithium acetate.

Mn (II) concentration in solution (Mn^{+2}), as a product of the reduction of Mn (IV) oxides (MnO_2), was comparable in the two systems. Mn reduction occurred prior to the test, a small increase was observed as the reducing front reached the observation points and slowly returned to pre-test conditions (Figure 5). Note that manganese reduction was observed in the same samples where denitrification was occurring. This is explained by 1) the potential coexistence of these two processes at similar redox potentials [8] or 2) the fact that water sampling was done by pumping, which implied flow average concentrations and a potential induced mixing of waters with different hydrochemistry.

Initial Fe concentrations in solution (assumed Fe^{+2}, given the neutral pH) were low and comparable to those of the inflow, suggesting that little, if any, iron reduction occurred during normal operation prior to the LiAc injection. Fe concentration grew at all observation points as the reducing front reached them, which indicates Fe (III) reduction, similar to the Mn reduction discussed above. Nevertheless, whereas Mn^{+2} concentration was stable in time, a decrease of Fe^{+2} concentration is observed in B2, with a tendency to the inflow concentration. This decrease of Fe^{+2} could be associated with potential precipitations of secondary iron minerals. Indeed, this is geochemical plausible since the saturation index of some sulfur minerals (e.g. pyrite and mackinawite) are higher than 0 (results not shown) and a similar behavior has been previously observed in similar environments[9].

Sulfate concentrations dropped after the reducing front reached every observation point (see Figure 4) but did not differ significantly from the inflow concentration. Thus, it might be conjectured that some sulfate reduction has occurred after the acetate injection, which would be consistent with the slight drop in Fe concentrations.

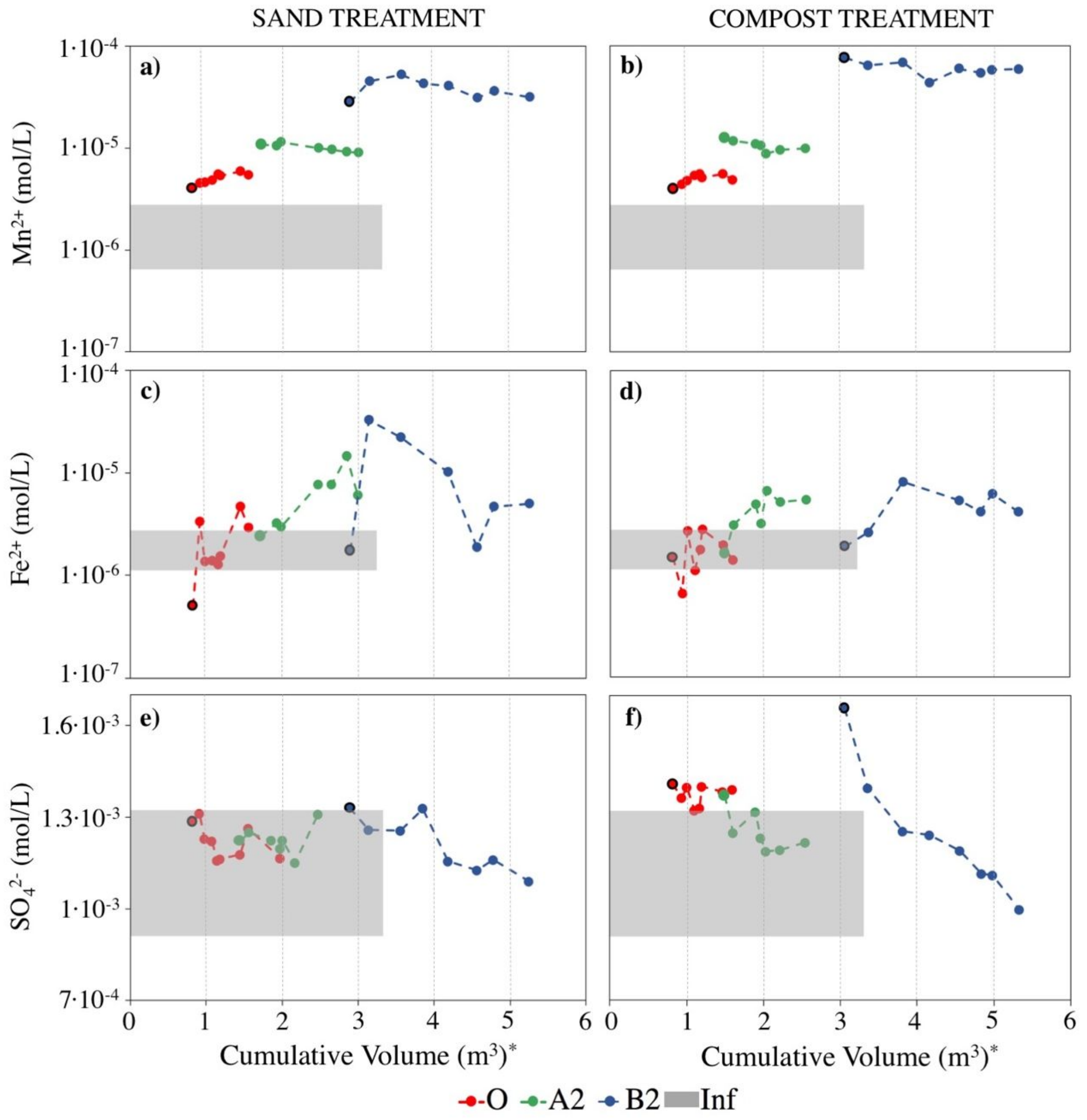

Figure S4. Evolution of the concentration of manganese (a,b), iron (c,d), and sulfate (e,f) *versus* the square root of the cumulative volume along the two SAT systems. The shadow rectangle represents the variability of concentration/value in the influent. The dots with the black border represent the prior conditions before the injection of lithium acetate.

S11. Water results for BP-4 and AVO

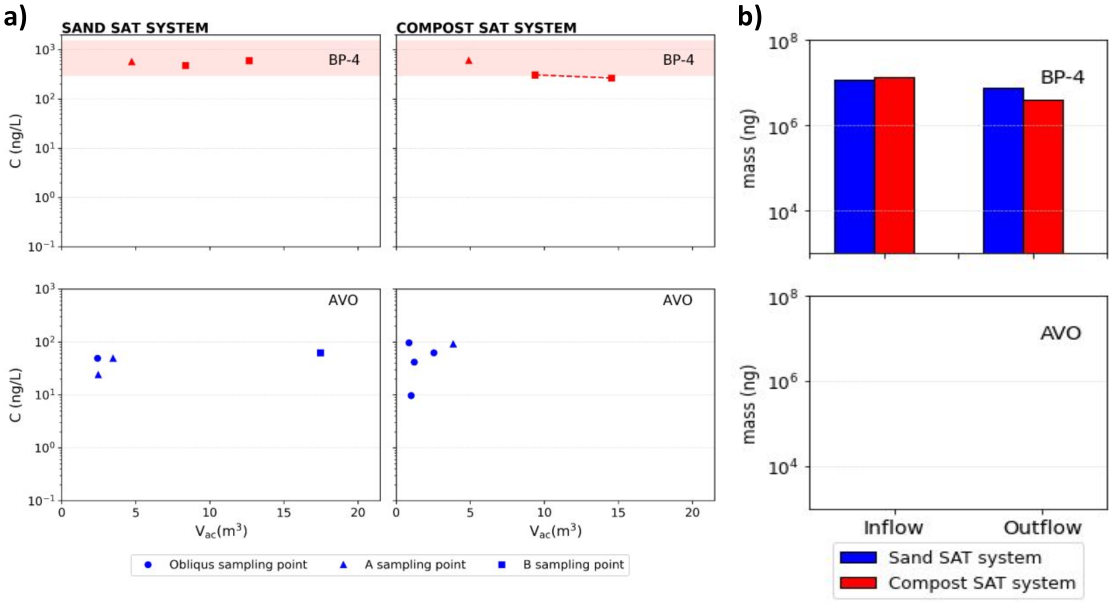

Figure S4. a) Evolution of the concentration of BP-4 and AVO at the different sampling points versus the cumulative volume along the two SAT pilot systems. The shadow rectangle represents the variability of concentration/value in the influent. b) Water mass balance of BP-3 and TPs in the two SAT systems. The "inflow" represents the cumulative mass input during the initial 12 days of the experiment, while the "outflow" means the mass present in the effluent after undergoing the residence time within the SAT system for the same duration.

S12. Biofilm characterization and fraction of organic carbon in the two SAT Systems

Table S10. UVFs concentration at the biofilm samples of the sand SAT system. UVFs concentrations are referred to g of dry aquifer sediment.

	EPS ($\mu g\ glucose\ /cm^3$)		Bacterial density ($cel \times 10^7/cm^3$)		f_{oc} (%)	
	ST	CT	ST	CT	ST	CT
Initial	32.29 ± 12.7[illegible]	27.01 ± 4.38	9.98 ± 1.65	13.49 ± 1.63	0.098 ± 0.[illegible]	0.14 ± 0.0[illegible]
After LiAc	23.11 ± 12.8[illegible]	13.65 ± 9.94	12.46 ± 5.12	9.41 ± 4.27		

S13. Mass balance of BP-3 and TP in ST SAT treatment

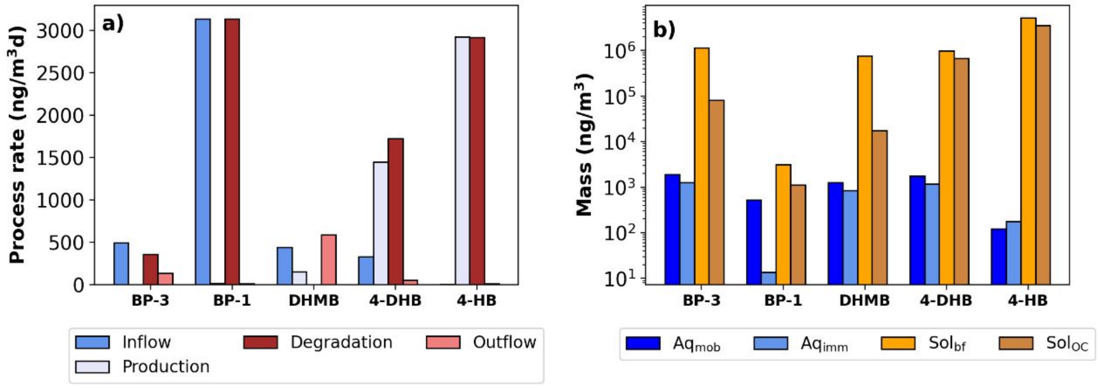

Figure S5. Summary of retention and degradation processes computed for BP3 and its TPs in the ST. a) Mass balance terms (ng/m3/day) for ST, with cold colors for inputs (inflow and production from parent compounds) and cold colors for outputs (outflow and degradation); and b) mass retained in the aqueous phase and solid phase (biofilm and aquifer sediments).

S13. References

(1) Valhondo, C.; Martínez-Landa, L.; Carrera, J.; Díaz-Cruz, S. M.; Amalfitano, S.; Levantesi, C. Six Artificial Recharge Pilot Replicates to Gain Insight into Water Quality Enhancement Processes. *Chemosphere* **2020**, *240*. https://doi.org/10.1016/j.chemosphere.2019.124826.

(2) Perujo, N.; Sanchez-Vila, X.; Proia, L.; Romaní, A. M. Interaction between Physical Heterogeneity and Microbial Processes in Subsurface Sediments: A Laboratory-Scale Column Experiment. **2017**. https://doi.org/10.1021/acs.est.6b06506.

(3) Gago-Ferrero, P.; Alonso, M. B.; Bertozzi, C. P.; Marigo, J.; Barbosa, L.; Cremer, M.; Secchi, E. R.; Azevedo, A.; Lailson-Brito, J.; Torres, J. P. M.; Malm, O.; Eljarrat, E.; Díaz-Cruz, M. S.; Barceló, D. First Determination of UV Filters in Marine Mammals. Octocrylene Levels in Franciscana Dolphins. *Environ. Sci. Technol.* **2013**, *47* (11), 5619–5625. https://doi.org/10.1021/es400675y.

(4) Sunyer-Caldú, A.; Diaz-Cruz, M. S. Development of a QuEChERS-Based Method for the Analysis of Pharmaceuticals and Personal Care Products in Lettuces Grown in Field-Scale Agricultural Plots Irrigated with Reclaimed Water. *Talanta* **2021**, *230*, 122302. https://doi.org/10.1016/J.TALANTA.2021.122302.

(5) Luther, G. W.; Sundby, B.; Lewis, B. L.; Brendel, P. J.; Silverberg, N. Interactions of Manganese with the Nitrogen Cycle: Alternative Pathways to Dinitrogen. *Geochim. Cosmochim. Acta* **1997**, *61* (19), 4043–4052. https://doi.org/10.1016/S0016-7037(97)00239-1.

(6) Yang, W. H.; Weber, K. A.; Silver, W. L. Nitrogen Loss from Soil through Anaerobic Ammonium Oxidation Coupled to Iron Reduction. *Nat. Geosci.* **2012**, *5* (8), 538–541. https://doi.org/10.1038/NGEO1530.

(7) Hu, B. L.; Shen, L. D.; Xu, X. Y.; Zheng, P. Anaerobic Ammonium Oxidation (Anammox) in Different Natural Ecosystems. *Biochem. Soc. Trans.* **2011**, *39* (6), 1811–1816. https://doi.org/10.1042/BST20110711.

(8) Stumm, W.; Morgan, J. J. *Aquatic Chemistry: Chemical Equilibria and Rates in Natural Waters*; 1996. https://doi.org/10.5860/choice.33-6312.

(9) Rodríguez-Escales, P.; Sanchez-Vila, X. Modeling the Fate of UV Filters in Subsurface: Co-Metabolic Degradation and the Role of Biomass in Sorption Processes. *Water Res.* **2020**, *168*, 115192. https://doi.org/10.1016/j.watres.2019.115192.
